# Interaction of camel Lactoferrin derived peptides with DNA: a molecular dynamics study

**DOI:** 10.1186/s12864-020-6458-7

**Published:** 2020-01-20

**Authors:** Zana Pirkhezranian, Mojtaba Tahmoorespur, Xavier Daura, Hassan Monhemi, Mohammad Hadi Sekhavati

**Affiliations:** 1 Department of Animal Science, Faculty of Agriculture, Ferdowsi University of Mashhad, Mashhad, Iran; 2 Institut de Biotecnologia i de Biomedicina, Universitat Autònoma de Barcelona, Bellaterra, Spain; 3 Catalan Institution for Research and Advanced Studies (ICREA), Barcelona, Spain; 4 Department of Chemistry, Faculty of Science, University of Neyshabur, Neyshabur, Iran

**Keywords:** Antimicrobial peptide, DNA binding, Lactoferrin, Molecular dynamics simulation, CLFchimera

## Abstract

**Background:**

Lactoferrampin (LFampin), Lactoferricin (LFcin), and LFchimera are three well-known antimicrobial peptides derived from Lactoferrin and proposed as alternatives for antibiotics. Although the intracellular activity of these peptides has been previously demonstrated, their mode of action is not yet fully understood. Here, we performed a molecular dynamics simulation study to understand the molecular interactions between camel Lactoferrin derived peptides, including CLFampin, CLFcin, and CLFchimera, and DNA as an important intracellular target.

**Results:**

Our results indicate that all three peptides bind to DNA, albeit with different propensities, with CLFchimera showing the highest binding affinity. The secondary structures of the peptides, modeled on Lactoferrin, did not undergo significant changes during simulation, supporting their functional relevance. Main residues involved in the peptide-DNA interaction were identified based on binding free energy estimates calculated over 200 ns, which, as expected, confirmed strong electrostatic interactions between DNA phosphate groups and positively charged peptide side chains. Interaction between the different concentrations of CLFchimera and DNA revealed that after binding of four copies of CLFchimera to DNA, hydrogen bonds between the two strands of DNA start to break from one of the termini.

**Conclusions:**

Importantly, our results revealed that there is no DNA-sequence preference for peptide binding, in line with a broad antimicrobial activity. Moreover, the results showed that the strength of the interaction between DNA and CLFchimera is concentration dependent. The insight provided by these results can be used for the rational redesign of natural antimicrobial peptides targeting the bacterial DNA.

## Background

Antibiotic resistance is becoming a serious global health problem, as infections by multidrug-resistant pathogens are increasing at an alarming pace. There is thus an urgent need to introduce new and safe antimicrobial agents, including antimicrobial peptides (AMPs), as alternatives to current antibiotics [1]. AMPs have evolved as a natural defense mechanism for fighting microbial infections [1]. They are a diverse group of innate immune system molecules that exist in all organisms [1]. AMPs usually contain 12–50 amino acid residues, have a net positive charge and an amphipathic structure [2–4]. One subgroup of AMPs includes peptides derived from large proteins. Lactoferrampin (LFampin) and Lactoferricin (Lfcin) are two well-known antimicrobial peptides derived from the Lactoferrin protein (LF) [5, 6]. These two cationic antimicrobial peptides have activity against a broad spectrum of microorganisms including bacteria, fungi and viruses [5, 6].

We have recently reported that a camel Lactoferrin chimera (CLFchimera) resulting from the fusion of the C-terminal ends of camel Lactoferricin 17–30 (CLFcin) and camel Lactoferrampin 265–284 (CLFampin) using the side chain of lysine as linker to the second peptide, has a broad-spectrum activity against both Gram-positive and Gram-negative bacteria [7–9]. Furthermore, Reyes-Cortes et al. (2016) showed that this chimeric peptide mediated its antibacterial activity by entering the cytoplasm through translocation across the bacterial membrane and possibly interacting with internal organelles [10]. To date, there has been no precise explanation for the mechanisms underlying the antimicrobial peptide function, but it is known that DNA is one of the most important intracellular targets for AMPs [11]. Thus, nucleic acids have been proven as intracellular targets for some antimicrobial peptides such as MDpep9 [11], Buforin I [12], Indolicidin [13], Cecropin PR39 [14], and NK18 [15]. Previous computational studies also showed that Buforin II (from the stomach tissue of the Asian toad *bufo garagrizans*) and Lasioglossin II (derived from bee venom) had considerable affinity for DNA [16, 17]*.* Considering these reports, Uyterhoeven et al. (2008) showed using MD simulation that Arg 2, Arg 14 and Arg 20 of Buforin II were mainly responsible for the interaction with DNA and using Fluorescent Intercalator Displacement (FID) assay indicated that disrupting Buforin-DNA interactions generally decreased the antibacterial activity of the peptide [16]. In another study, Tang et al. (2009) demonstrated that MDpep9, a recently discovered antimicrobial peptide derived from larvae of housefly (*Musca domestica*), a traditional food source in China, is able to form bonds with DNA phosphate groups and insert between the base pairs of the DNA helix [11].

Although the intracellular activity of Lactoferrin derived peptides has been previously demonstrated [10], the exact mechanism of action has not been yet established. As recognition of specific DNA sequences by proteins is highly complex, involving structural, energetic and dynamic aspects, the interaction cannot be easily characterized at the atomic level by experimental approaches alone [18]. The use of computational techniques such as molecular dynamics (MD) simulations provides complementary information, inaccessible experimentally, which relates directly to the thermodynamics and kinetics of the system. Herein, homology-based models were constructed for camel LFcin, LFampin and LFchimera and their interaction with DNA was analyzed using MD simulation, as a means to understand a reported intracellular mechanism of action of these peptides. The findings of this study provide basic directions for future studies regarding the function of AMPs with intracellular activity and their potential redesign with therapeutic purposes.

## Methods

### Molecular structure models

An arbitrary 12-bp DNA sequence adopting a canonical BDNA structure (entry 1BNA from the Protein Data Bank) was initially chosen for this study (Table 1). Since the simulations using this sequence indicated that the interaction with the peptides is DNA-sequence independent (through the backbone phosphates), no additional sequences were used in the study. The length of the DNA allows the interaction with more than one peptide, as shown in the Results and Discussion. The native structure of camel Lactoferrin was also retrieved from the PDB (entry 1DTZ). Camel Lactoferrin was used as a template structure for peptide modeling. Lactoferricin, Lactoferrampin and CLFchimera were modeled with Modeller 9.2 [19] and PEP-fold server (http://bioserv.rpbs.univ-paris-diderot.fr/services/PEP-FOLD/) [20] using default parameters. The quality of the models was examined with PROCHECK (http://servicesn.mbi.ucla.edu/PROCHECK/) [21].

**Table 1:** Details of sequences, simulation lengths and replicates

System	Composition	Simulation Length (ns)	Replicates	Box size (nm^3^)
BDNA	CGCGAATTCGCG	200	3	4.28
CLFampin	DLIWKLLVKAQEKFGRGKPS	200	3	4.98
CLFcin	KKCAQWQRRMKKVR	200	3	4.89
CLFchimera	DLIWKLLVKAQEKFGRGKPS KRVKKMRRQWQACKKS	200	3	5.19
1-CLFampin / BDNA		200	3	6.65
1-CLFcin / BDNA		200	3	6.38
1-CLFchimera / BDNA		200	3	6.90
2-CLFchimera / BDNA		200	3	7.81
3-CLFchimera / BDNA		200	3	8.36
4-CLFchimera / BDNA		200	3	8.96

### Molecular dynamics simulations

The complexes BDNA-CLFampin, BDNA-CLFcin, and BDNA-CLFchimera were studied by molecular dynamics simulation with the GROMACS 2016.1 package [22–24] and CHARMM27 force field [25]. Peptides and DNA were solvated in a cubic box using the Simple Point Charge (SPC) water model [26]. To neutralize the overall charge of the systems, Na and Cl ions were added as appropriate. Periodic boundary conditions were applied from this step onward. The system was first energy minimized using the steepest descent algorithm to relax high-energy contacts. After energy minimization, the system was simulated under the NPT ensemble for 500 ps, with initial velocities taken from a Maxwell-Boltzmann distribution corresponding to 100 K. During this initial simulation time, the peptide and DNA atoms were positionally restrained while the temperature was gradually increased from 100 K to 300 K at 1 atm. Bond lengths were constrained for all atoms using the LINCS algorithm (SETTLE for water), allowing a time step in the leap-frog integrator of 2 fs. Temperature and pressure were couple to the reference values using the Nosé-Hoover and Parrinello-Rahman algorithms, respectively [27–29]. Additional 100 ps at 300 K and 1 atm, without position-restraints, were subsequently run. In the production phase, the equilibrated systems were run in the NPT ensemble at 1 atm and 300 K for 200 ns. Long-range electrostatics were evaluated using the Particle Mesh Ewald (PME) algorithm [28]. The real space component of PME and the van der Waals interactions were calculated with a cutoff of 1.0 nm. Three replicates of 200 ns were run per system, with different initial configurations generated by insertion of the peptides at random positions. The simulations performed and their lengths are detailed in Table 1. Dynamics and stability of each peptide and BDNA, including root mean square deviation (RMSD), root-mean-square-fluctuations (RMSF), solvent accessible surface area (SASA), contacting surface area (CSA), hydrogen bonds, salt bridges, and center of mass distance were analyzed during the simulation using GROMACS built-in tools. An RMSD-based conformational clustering algorithm, using the gmx-cluster module of GROMACS, was applied to extract representative structures. The clusters were obtained using a cut-off of 1.5 Å for the RMSD to the centroid.

### Binding free energy estimates

Binding free energies were estimated for BDNA-CLFampin, BDNA-CLFcin, and BDNA-CLFchimera complexes using molecular mechanics energies in combination with Poisson-Boltzmann and surface area continuum solvation (MM/PBSA). The calculations were performed with the g_mmpbsa program (https://rashmikumari.github.io/g_mmpbsa/) [30], using the single trajectory approach. The solute dielectric constant was set to 8 [31] and the ionic strength was chosen to correspond to a NaCl concentration of 150 mM. The calculation of the G_polar_ solvation term was performed with the linearized Poisson-Boltzmann (PB) equation using a grid resolution of 0.05 nm and the *bondi* set of atomic radii. The G_nonpolar_ term was calculated with the SASA model using default parameters [30]. The entropic component of the binding free energy was disregarded. The average binding energy and its standard deviation were calculated with the MmPbSaStat.py python script (http://rashmikumari.github.io/g_mmpbsa/) using the second half of the simulations production phase (100 to 200 ns), by taking 1000 snapshots at 100-ps intervals. To estimate the contribution of each residue to the total binding free energy, the MmPbSaDecomp.py python script was used [30, 32]. It should be noted that this approach represents a crude estimate of the binding free energy that, most certainly, severely overestimates the real value, as noted by several authors [33]. However, the limitations of the approach are likely to affect the related systems studied here in similar ways and are therefore expected to allow for a qualitative comparison.

## Results and discussion

### Molecular dynamics simulation in aqueous solution of the isolated peptides and BDNA

Before simulating the interaction between the different peptides and BDNA, the individual model structures were relaxed along independent 200-ns simulations, performed in triplicate (Table 1). To that end, the homology models obtained for the peptide structures were first examined for overall quality. The Ramachandran plot for CLFampin, CLFcin and CLFchimera revealed that 93.3, 100.0 and 93.5% of the residues were situated within the most favored region, respectively, whereas the remaining residues were found within the additional allowed region.

### Structural fluctuation analysis

**Fig. 1:**
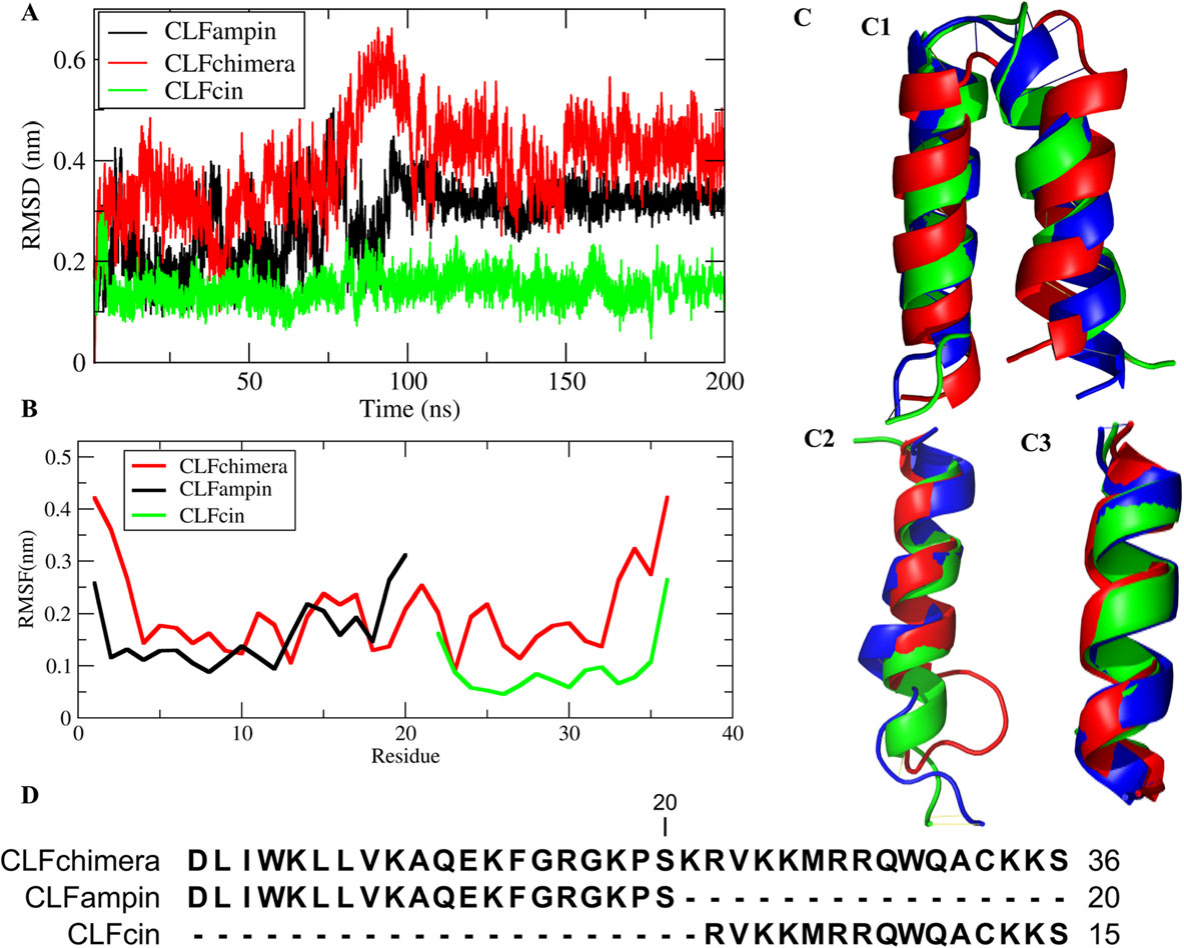
Structural fluctuation analysis. **a** RMSD as a function of time; **b** RMSF per residue; **c** Cartoon structure of CLFchimera (C1), CLFampin (C2) and CLFcin (C3) at 0, 100 and 200 ns (red, blue and green, respectively); **d** Sequence alignment of the three peptides. RMSD and RMSF quantities were computed for structures at 0.1-ns intervals from the 200-ns simulations after least square fitting to the initial structure using the backbone atoms

Root-mean-square deviations from the initial structure of the peptide as a function of simulation time and root-mean-square fluctuations of peptide residues are presented, for one of the 200-ns replicates, in Fig. 1. The behavior of these quantities in the remaining replicates is consistent with the observations made here (see Additional file 1: Figure S1 and Additional file 2: Figure S2). The RMSD values are stable after the initial 100 ns, the larger peptide CLFchimera showing higher RMSD and RMSF values. CLFchimera was obtained from the C-term-C-term fusion of CLFampin 265–284 and CLFcin17–30, using a lysine (Lys21) as linker [7, 8]. Figure 1b shows that the global fluctuations of the corresponding sequences in the shorter peptides are lower in general than in the fusion peptide, as expected in light of the structures shown in Fig. 1c. It is worth noting that the shorter CLFcin adopts a more stable helical structure than CLFampin when isolated in solution, to become more flexible in the fusion peptide. Structures from the stable part of the 200 ns simulations with all residues in the most favored regions of the Ramachandran plot were used as initial structures for the corresponding simulation of peptide-DNA systems.

### Molecular dynamics simulation of the peptide-DNA systems

Simulations between CLFchimera, CLFcin and CLFampin and BDNA were performed for 200 ns in triplicate. To construct the system, the peptide was introduced in the BDNA box at a random position and orientation. Center of mass distance (COM), hydrogen bonds, salt bridges and contacting surface area between peptide and DNA were analyzed.

### Center of mass distances

**Fig. 2:**
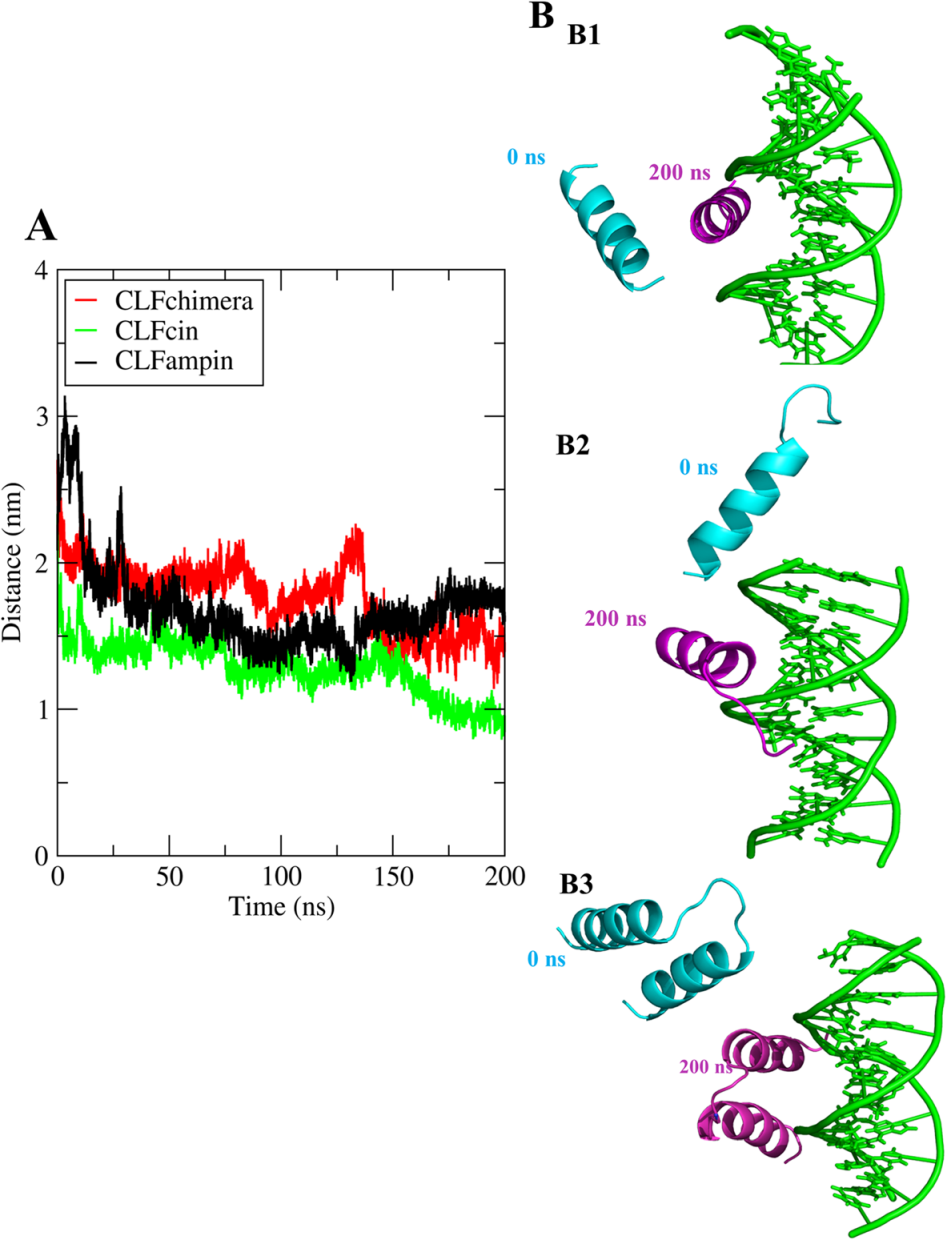
COM distance analysis. **a** COM distances between CLFcin, CLFampin and CLFchimera and DNA along 200 ns. **b** Structures at times t = 0 (cyan) and t = 200 ns (purple): (B1) CLFcin-DNA, (B2) CLampin-DNA, and (B3) CLFchimera-DNA

The center of mass distance between peptide and DNA was calculated as a function of time (Fig. 2a). Side view of snapshots of the first and last configurations are shown in Fig. 2b. In all three replicates, COM distances were initially around 3, 3 and 4 nm for CLFcin, CLFampin and CLFchimera, respectively. The peptides instantly moved toward the DNA grooves and COM distances decreased rapidly. The three replicates show some differential behavior in terms of final distance and convergence (Additional file 3: Figure S3A and Additional file 4: Figure S4A), as well as in terms of position and orientation (Additional file 3: Figure S3B and Additional file 4: Figure S4B), suggesting that the binding is not specific, as demonstrated further below.

### Number of hydrogen bonds and salt bridges

**Fig. 3:**
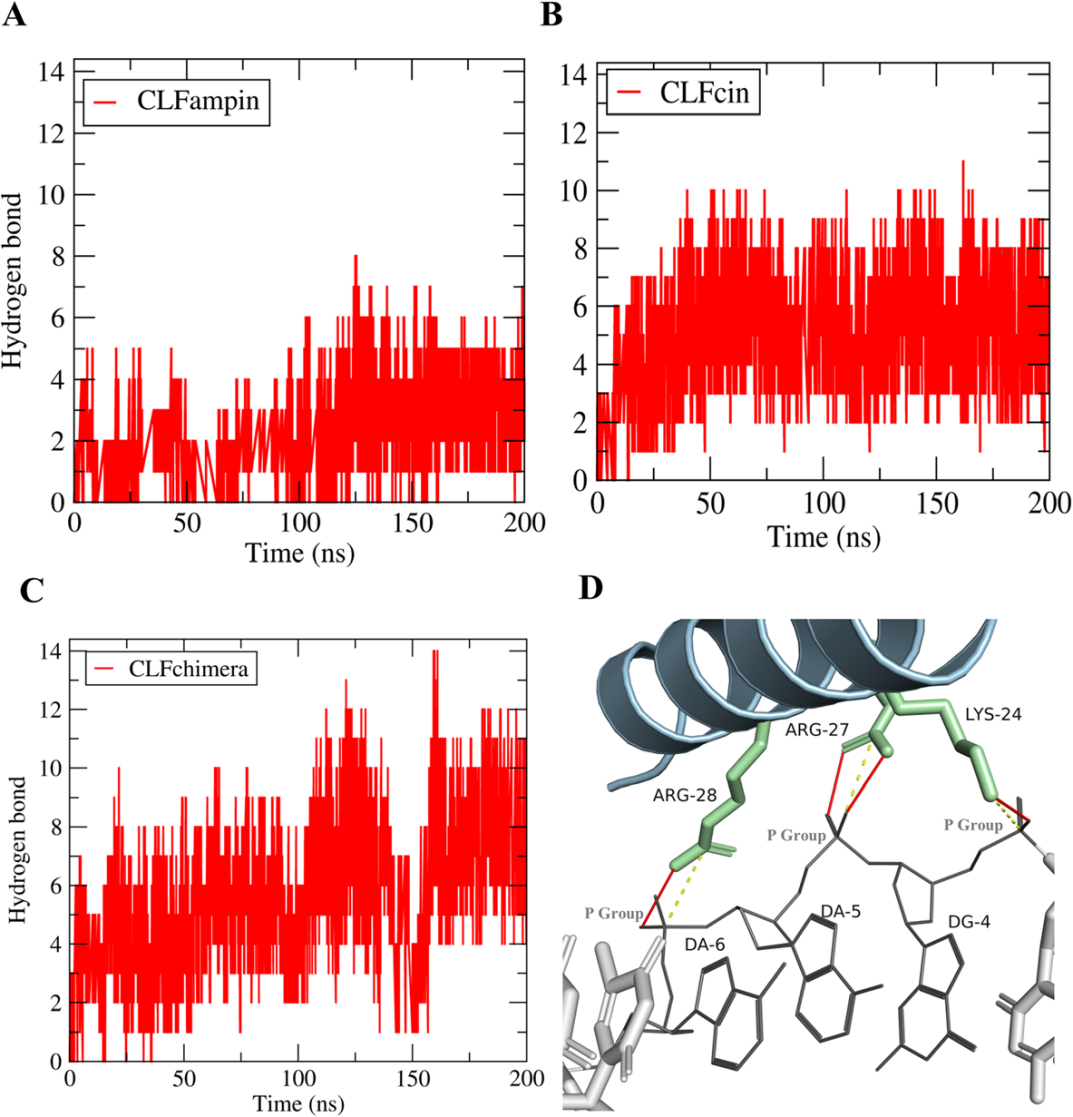
Number of hydrogen bonds with DNA as a function of simulation time (200 ns). **a** CLFampin, **b** CLFcin, **c** CLFchimera. **d** Snapshot at *t* = 135 ns of the CLFchimera-DNA system, indicating hydrogen bonds (red lines) and salt bridges (yellow dashed)

The number of hydrogen bonds between peptide and DNA showed significant variation during simulation (Fig. 3; Additional file 5: Figure S5 and Additional file 6: Figure S6). The average number of hydrogen bonds in the second half of the three simulation replicates (100–200 ns, 300 ns in total) was 5.66 ± 0.23, 4.61 ± 0.55 and 2.63 ± 0.27 for CLFchimera, CLFcin and CLFampin, respectively (see also Additional file 7: Table S1 for details), suggesting that CLFchimera establishes more stable interactions with DNA.

A representative snapshot of the CLFchimera-DNA interaction is illustrated in Fig. 3d. In this frame, it can be seen that hydrogen-bonding interactions are mainly established between positively charged residues of the peptide and the DNA-backbone phosphate groups, which constitute also salt bridges.

Salt bridges also play a fundamental role in protein-ligand interactions [34, 35]. In several studies, a cutoff of 4 Å between N-O atom pairs has been used to define salt bridge formation [36, 37]. Here, we calculated salt bridges between P atoms from the nucleic-acid backbone and N atoms from Lysine and Arginine residues, and thus used 5 Å as cutoff. The average number of salt bridges in the second half of the three simulation replicates between DNA and CLFchimera, CLFcin, CLFampin were 4.09 ± 016, 3.17 ± 0.28 and 1.71 ± 0.44 (see Additional file 7: Table S1 for details). Again, CLFchimera establishes more salt bridges with DNA than the other two peptides.

### Contacting surface area

**Fig. 4:**
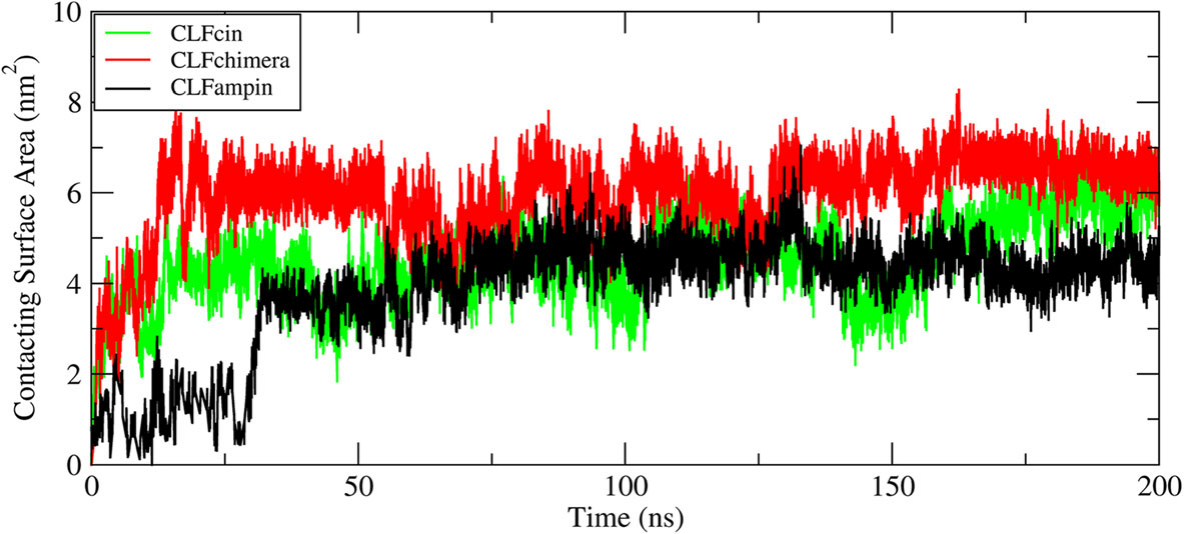
Contacting surface area between peptide and DNA along a 200 ns MD simulation

The solvent-accessible surface area was calculated with the Gromacs library [38]. The contacting surface area can be then calculated using the following formula: *CSA = (SASA Peptide(s) + SASA DNA – SASA Peptide(s)-DNA)/2* [39]. Initially, the CSA was close to zero due to the distance between peptides and DNA. The evolution of the CSA is shown in Fig. 4 for one of the simulation replicates (see Additional file 8: Figure S7 for the other two). In all three replicates, the CSA is stable after the initial 100 ns, indicating a stable interaction has been reached. The average CSA in the period 100–200 ns is 5.92 ± 0.41, 4.9 ± 0.1, and 4.76 ± 0.36 nm^2^ for the CLFchimera, CLFcin and CLFampin systems, respectively (see Additional file 7: Table S1 for details). The CSA is higher for CLFchimera than for the other two peptides, in line with the observed interactions.

### MM/PBSA binding free energy estimate

**Fig. 5:**
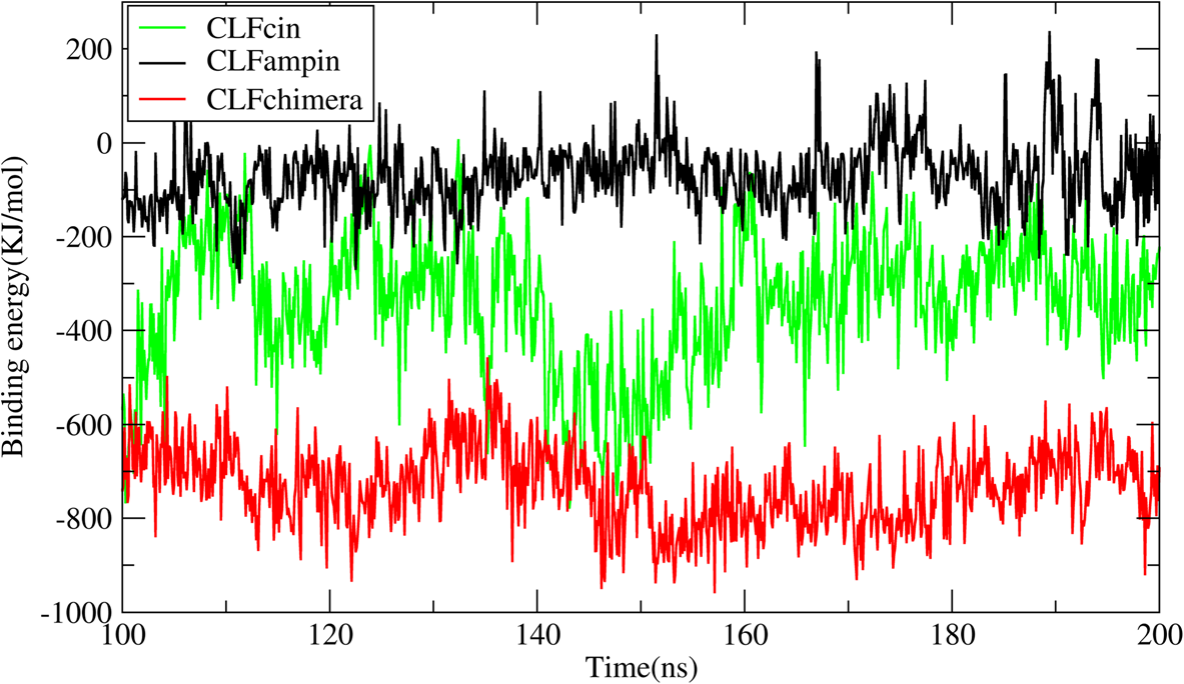
Estimated binding free energy for the peptide-DNA systems. Calculated with the MM/PBSA method on the 100–200 ns period of one of the simulation replicates

The binding free energy was estimated using the MM/PBSA method. The results for the period 100–200 ns in one of the replicates are presented in Table 2. As indicated in the Methods section, particularly for this type of systems (high charge density), the single-trajectory MM/PBSA approach represents a very crude estimate of the binding free energy that, most certainly, severely overestimates the real value. Nevertheless, the calculations will be used here to qualitatively compare and rank the different systems, which should be relatively safe given that the nature of the interactions is the same in all cases. The results indicate that CLFchimera has the lowest DNA-binding energy. The plot of the binding free energy along the period 100–200 ns in one of the replicates is shown in Fig. 5 (see Additional file 9: Figure S8 for the other two replicates). No significant differences in the obtained binding free energy values were observed among replicates (− 786 ± 2.545, − 731 ± 3.521 and − 712 ± 7.801 kJ/mol for CLFchimera; − 340 ± 4.437, − 352 ± 4.437 and − 316 ± 7.215 kJ/mol for CLFcin; − 71 ± 3.063, − 78 ± 5.103 and − 62 ± 2.202 kJ/mol for CLFampin).

**Table 2:** Binding free energy for the three peptide-DNA systems calculated by the MM/PBSA method (one simulation replicate)

Peptides	van der Waal (kJ/mol)	Electrostatic (kJ/mol)	Polar solvation (kJ/mol)	Non-Polar solvation (kJ/mol)	Binding energy (kJ/mol)
CLFcin	− 141 ± 1	− 1885 ± 2	1707 ± 7	−21 ± 0.1	−340 ± 4
CLFampin	− 120 ± 1	− 825 ± 1	891 ± 3	−18.1 ± 0.1	−71 ± 3
CLFchimera	− 152 ± 1	− 2396 ± 2	1781 ± 3	−20.75 ± 0.1	−786 ± 3

**Fig. 6:**
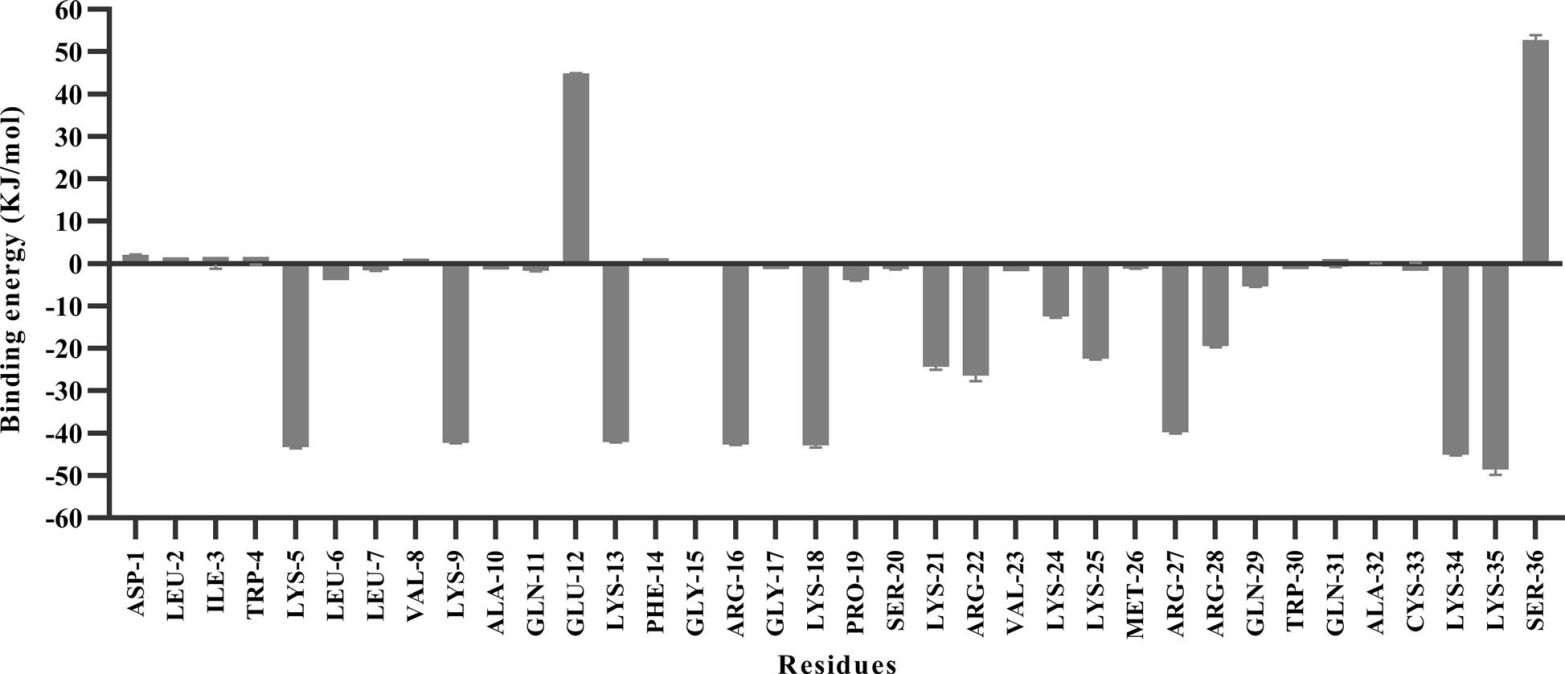
Contribution to DNA binding free energies of amino-acid residues in CLFchimera

The free energy values for the CLFchimera-DNA system were decomposed into residue contributions using the MmPbSaDecomp.py python script. The results, presented in Fig. 6 for one of the simulation replicates, indicate that residues LYS5, LYS9, LYS13, ARG16, LYS18, ARG27, LYS34 and LYS35 are more relevant for binding. On the other hand, GLU12 and SER36 have a detrimental effect. The contributions in the other two simulation replicates follow the same trends (Additional file 10: Figure S9).

Previous experimental studies revealed that substitution of positively charged residues such as LYS269, LYS277 and LYS282 with alanine in bovine Lactoferrampin (LYS9, LYS13 and LYS18 in CLFchimera) resulted in a dramatic decrease in antimicrobial activity [40, 41], a finding consistent with our in silico results (Fig. 6). However, Karn et al. (2006) showed that substitution of GLU276 (GLU12 in CLFchimera) with glycine in bovine Lactoferrampin had no effect on increasing antimicrobial activity [40]. Several experimental studies regarding bovine Lactoferricin indicated that the core hexapeptide “RRWQWR” in this peptide has a significant role in antimicrobial activity [42]. The first two amino acids from this central core in CLFchimera (ARG27 and ARG28) made a considerable contribution to the interaction with DNA in our simulations (Fig. 6); however, they were not as effective as other positively charged residues. Investigation of minimum distances (averaged over the three replicates) showed that LYS5 and LYS35 were closest to DNA, 0.13 ± 0.03 nm and 0.12 ± 0.02 nm, respectively (see Additional file 11: Figure S10).

As shown in Fig. 6, GLU12 and SER36 play a major inhibiting role in the interaction with DNA. Additional file 10: Figure S9 shows that they displayed also the largest minimum distance to DNA, with 0.64 ± 0.13 nm and 0.57 ± 0.09 nm, respectively.

### Effect of CLFchimera concentration on DNA binding

Based on the previous sections, CLFchimera showed a considerably higher affinity than CLFcin and CLFampin for DNA. Therefore, we chose the former peptide as candidate and evaluated the effect of its concentration on DNA binding by performing simulations with 1, 2, 3 and 4 CLFchimera molecules and one DNA helix.

### Number of hydrogen bonds and salt bridges

**Fig. 7:**
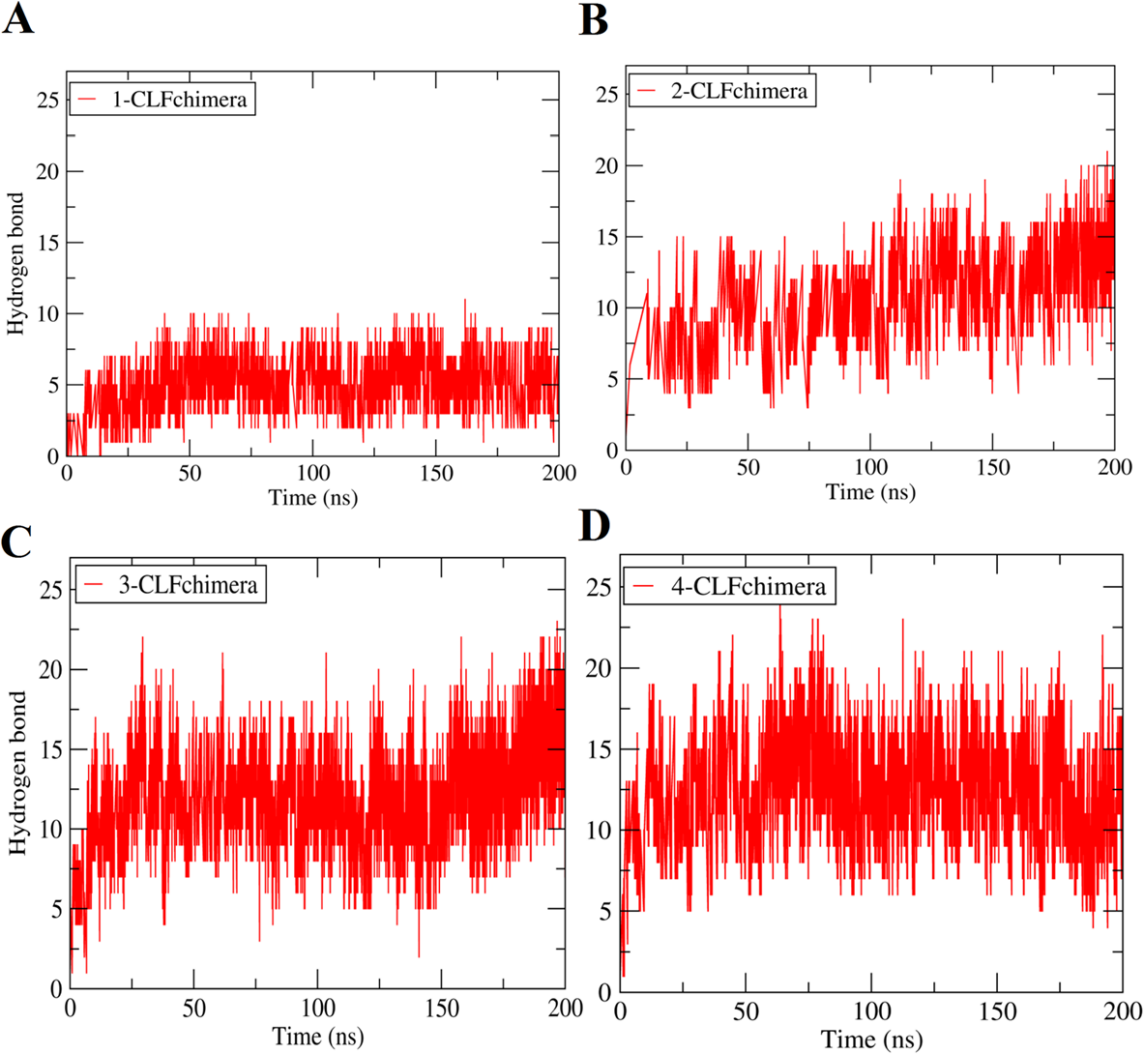
Number of hydrogen bonds with DNA at different concentrations of CLFchimera. **a** 1-CLFchimera, **b** 2-CLFchimera, **c** 3-CLFchimera, **d** 4-CLFchimera

As expected, when peptide concentrations rose, the number of hydrogen bonds between DNA and peptides increased but showed saturation (Fig. 7 for one of the replicates of each system; Additional file 12: Figure S11 and Additional file 13: Figure S12 for the other two replicates). The average number of hydrogen bonds in the 100–200 ns period of the three replicates was 5.66 ± 0.23, 9.66 ± 0.56, 11.75 ± 0.45, and 12.4 ± 0.37 for 1, 2, 3 and 4 peptides, respectively (see Additional file 7: Table S1 for details). The corresponding average number of salt bridges was 4.09 ± 0.16, 5.79 ± 0.72, 6.50 ± 0.24, and 7.03 ± 0.06, respectively (see Additional file 7: Table S1 for details). The largest change in the number of hydrogen bonds and salt bridges occurs in the transition between one and two peptides, and shows saturation after three peptides.

The average percentage of hydrogen bonds involving the DNA phosphate group revealed that nearly all hydrogen bonding was formed between peptide side chains and DNA phosphate groups (see Additional file 14: Table S2). As these groups are equal in all nucleic-acid bases, we may conclude that the binding of CLFchimera to DNA is DNA-sequence unspecific. For this reason, a potential DNA-related antimicrobial activity of this peptide could consist in the disruption of replication. Uyterhoeven et al., 2008 reported that Buforin II, another antimicrobial peptide, would probably target nucleic acids in a non-sequence-specific manner [16]. Sim et al., 2017 also examined the interaction of Buforin II and DesHDAP1 with DNA and suggested that a large percentage of hydrogen bonds were formed between peptide side chains and phosphate groups in the nucleic acid backbone (96.3 and 81.7%, respectively). Then, they demonstrated experimentally that Buforin II and DesHDAP1 did not show signs of sequence-specific DNA binding [43]. In another study on a particular AMP derived from Chinese traditional edible housefly larvae by Tang et al., 2009, it was revealed that the phosphate anion of the DNA double helix is one of the binding sites in DNA-peptide interaction [11]. The results of the present study are in accordance with all these previous findings. On the other hand, an experimental study on the interaction between bovine Indolicidin and DNA demonstrated that Indolicidin bound tightly to the ds [AT], ds [GC] and ds [AG] sequences, but formed loose bonds with ds [GT] [44].

### Contacting surface area

**Fig. 8:**
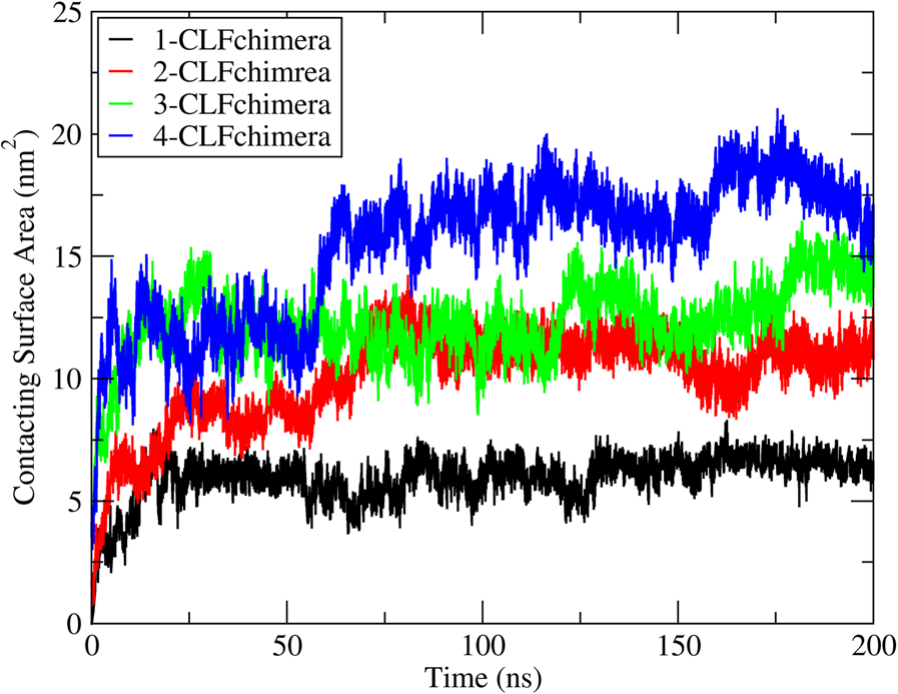
Contacting surface area at different concentrations of CLFchimera

Results from the CSA analysis at different concentrations of CLFchimera revealed an increase in CSA with peptide concentration (Fig. 8 for one of the replicates of each system and Additional file 15; Figure S13 for the other two replicates). The average CSA (three replicates) for 1, 2, 3 and 4 peptides was 5.92 ± 0.41, 10.30 ± 0.83, 11.79 ± 1.14 and 14.55 ± 1.39 nm^2^, respectively (see Additional file 7: Table S1 for details). The highest increase in CSA occurs for the transition between one and two peptides. However, the difference between 3 and 4 peptides is slightly larger than could be expected from the previous analysis of interactions, indicating that the increase in contact area is not proportionally translated in additional specific interactions.

### MM/PBSA binding free energy estimate

As shown in Table 3 (one of the replicates), the CLFchimera-DNA binding energy decreases with increasing peptide concentration. The decrease in binding energy per peptide is again largest in the transition from one to two peptides. The results for the different replicates are in this regard consistent: − 946 ± 4.504, − 927 ± 9.31 and − 894 ± 5.056 kJ/mol for 2-CLFchimera; − 1004 ± 4.007, − 1020 ± 9.23 and − 975 ± 8.09 kJ/mol for 3-CLFchimera; − 1027 ± 15.718, − 1071 ± 12.21 and − 1022 ± 10.12 kJ/mol for 4-CLFchimera. The saturation effect observed on going from three to four peptides could be partially due to repulsion between the highly charged peptides, in addition to crowding at the sites favorable for the interaction with DNA phosphate groups.

**Table 3:** Binding free energy estimated by the MM/PBSA method, for one replicate at each CLFchimera concentration

Peptides	van der Waal (kJ/mol)	Electrostatic (kJ/mol)	Polar solvation (kJ/mol)	Non-Polar solvation (kJ/mol)	Binding energy (kJ/mol)
1-CLFchimera	−152 ± 1	−2396 ± 2	1782 ± 3	−20.75 ± 0.1	−786 ± 3
2-CLFchimera	− 176 ± 1	− 2398 ± 3	1650 ± 7	−21.6 ± 0.1	−946 ± 4
3-CLFchimera	− 137 ± 2	− 2171 ± 6	1323 ± 10	−17.5 ± 0.2	−1004 ± 4
4-CLFchimera	−137 ± 4	− 2001 ± 11	1137 ± 24	−36 ± 0.5	−1027 ± 16

**Fig. 9:**
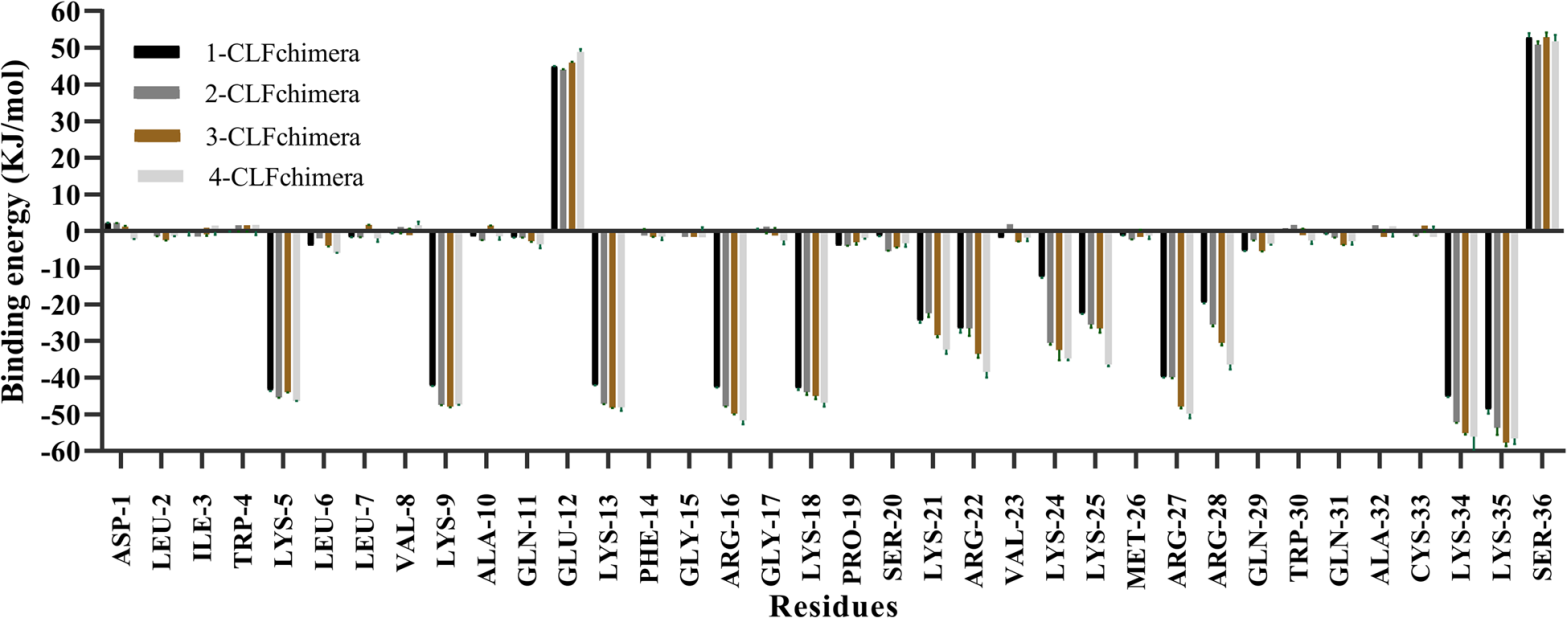
Contribution of amino-acid residues to DNA-binding energy at different concentrations of CLFchimera

The binding energy contribution per amino-acid residue (Fig. 9 for one replicate and Additional file 16: Figure S14 for the other two) indicates that the residues with important contributions are the same at the different concentrations. It also shows that they are primarily responsible for the decrease in binding energy with increasing concentration.

Tables 2 and 3 demonstrate that complex formation and stability was highly correlated with electrostatic interaction, as expected. Our MM/PBSA results conform to the results obtained by Pandey et al., 2018 on similar systems [18].

**Fig. 10:**
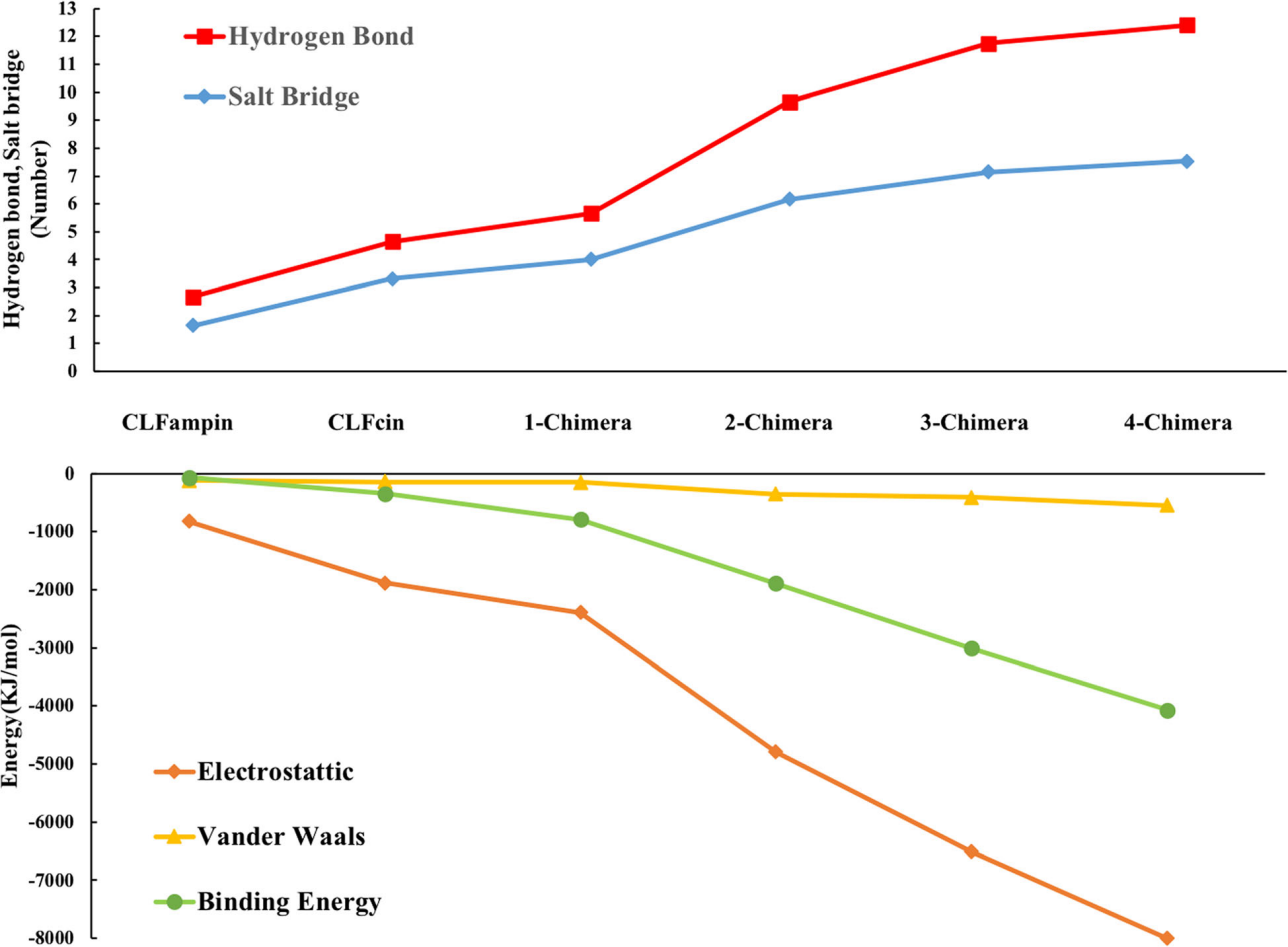
Trends in the number of hydrogen bonds and salt bridges, and electrostatic, van der Waals and binding energy in simulations of DNA with CLFampin, CLFcin and different concentrations of CLFchimera

A computational study by Khabiri et al., 2017 reported that changes in binding free energy do not correlate strongly with salt bridges or hydrogen bonding in protein-DNA interactions [45]. Contrary to their results, we show that for our peptide-DNA systems the electrostatic energy and binding free energy are correlated with the number of hydrogen bonds and salt bridges (Fig. 10), with an R^2^ between binding free energy and the number of hydrogen bonds and salt bridges of − 0.92 and − 0.95, respectively, and an R^2^ of − 0.95 and − 0.97 between electrostatic energy and hydrogen bonds and salt bridges, respectively.

### Structural characterization of CLFchimera

**Fig. 11:**
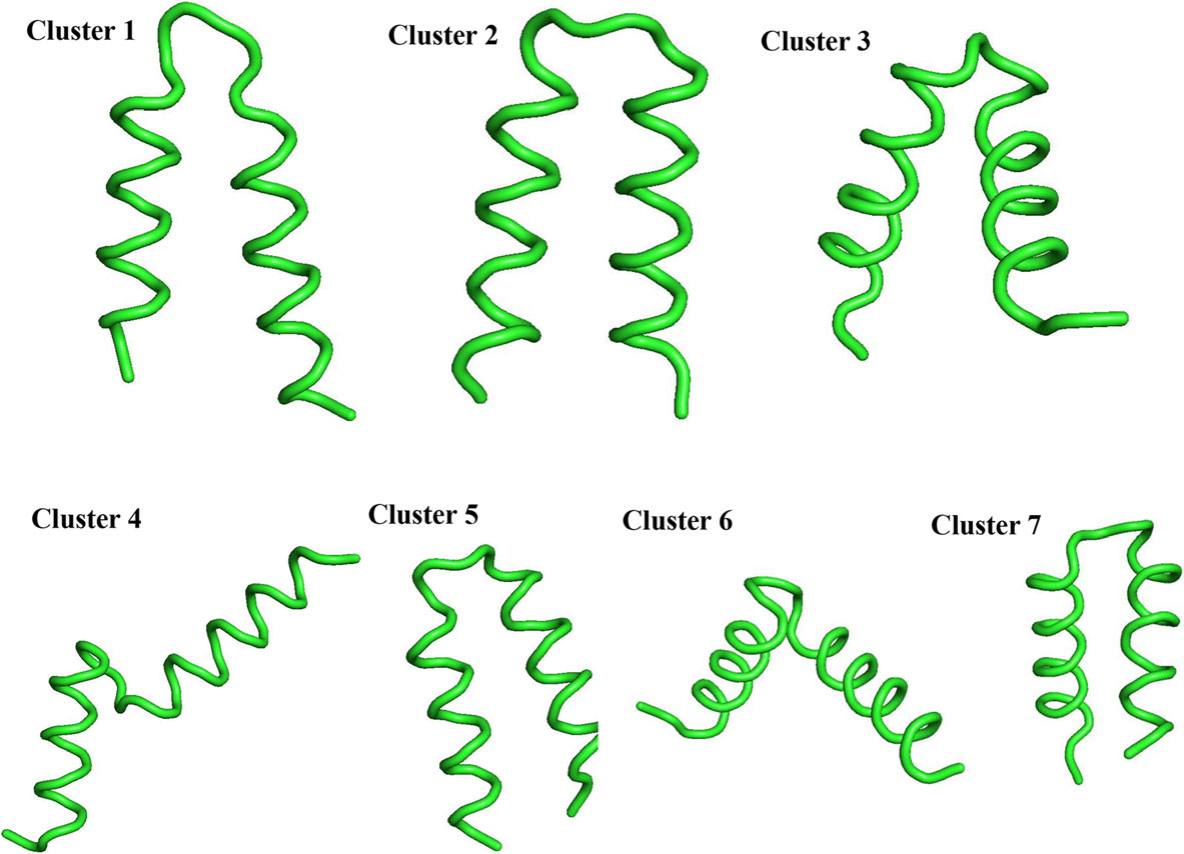
Top seven, most-populated conformations of CLFchimera in the simulations

To assess the conformational heterogeneity of the peptide, clustering analysis was performed for all simulation trajectories. CLFchimera was thus distributed into 22 clusters. The conformation of the top seven clusters, covering 97% of all structures, is shown in Fig. 11. Clusters one and two comprised 65% of all structures. The analysis shows that the helical segments of CLFchimera are stable along the simulations, with major conformational changes affecting the turn between them.

### Effect of CLFchimera concentration on DNA conformation

**Fig. 12:**
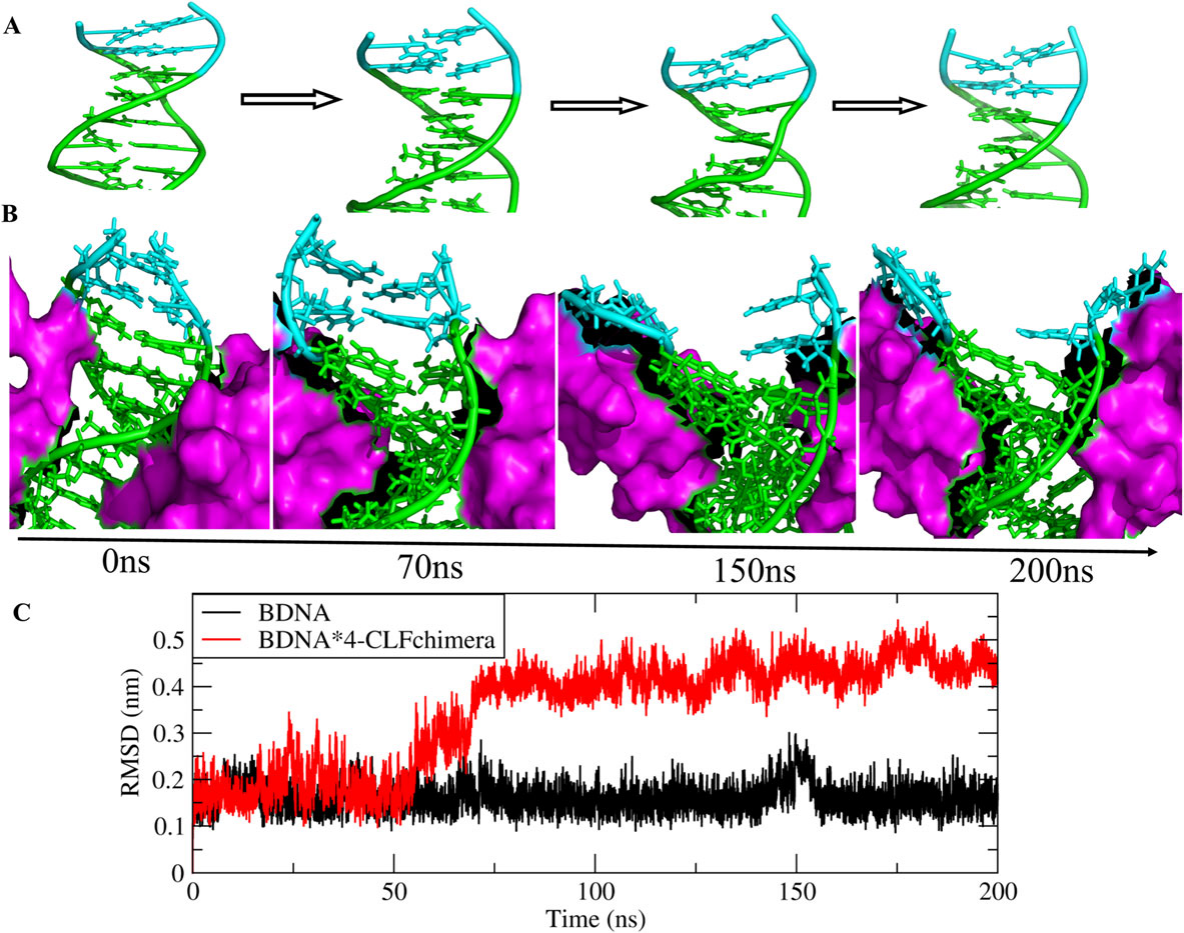
Monitoring of BDNA conformation during MD simulation. **a** changes in BDNA conformation in the absence of the peptide. **b** change of BDNA conformation with presence of four copies of the peptide. **c** RMSD analysis of the DNA without and with four copies of the peptide

**Fig. 13:**
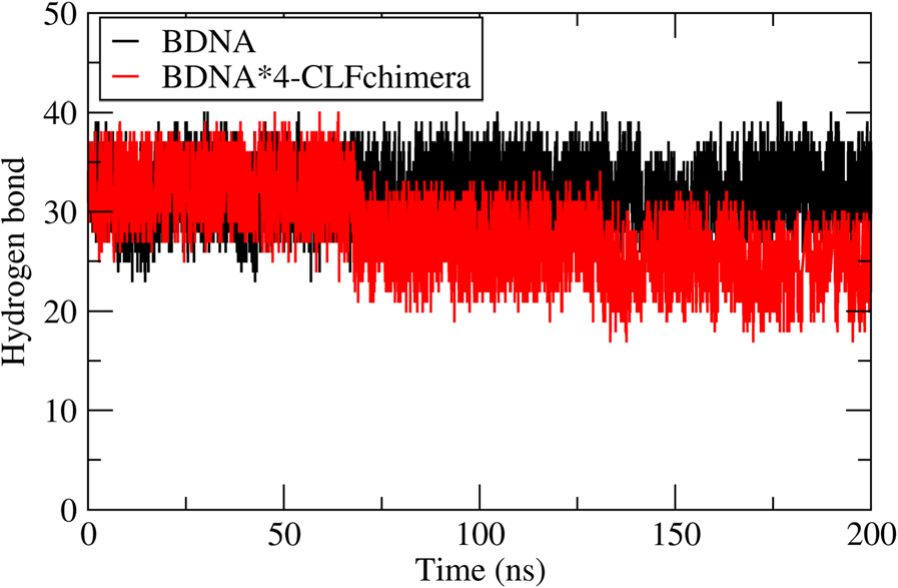
Analysis of the number of DNA interstrand hydrogen bonds in the presence of zero and four copies of CLFchimera

Analysis of RMSD and hydrogen bonds between the two strands of the DNA revealed that the presence of 1, 2 and 3 molecules of CLFchimera had no significant effect on DNA conformation during MD simulations. However, four copies of the peptide caused DNA partial denaturation (Fig. 12a and b), significantly increasing the RMSD values (Fig. 12c) and decreasing the number of backbone hydrogen bonds (Fig. 13) after about 50 ns of simulation. This result was observed for two (out of three) replicates of four copies of concentrations.

## Conclusions

To improve our understanding of the possible intracellular mechanisms of antimicrobial peptides (AMPs) derived from camel lactoferrin, we planned an in silico study based on molecular dynamics simulation. In this study, DNA was selected as a well-known intracellular target for AMPs. Overall, the simulation results indicated that the chimeric peptide CLFchimera has a higher affinity for DNA than its component peptides CLFcin and CLFampin. It is shown that the interaction between DNA and CLFchimera follows a saturation curve with increasing peptide concentration, the largest gain in binding free energy corresponding, for a 12-bp DNA, to the transition between one and two peptide copies. Saturation is reached after three peptide copies, with four copies inducing the denaturation of the DNA helix.

Binding free energy analysis revealed that the number of lysine and arginine residues (12 in CLFchimera, 4 in CLFampin, 7 in CLFcin) is largely responsible for the higher affinity of the CLFchimera-DNA complex. It is in fact concluded that electrostatic interactions and particularly salt bridges play a pivotal role in the interaction between CLFchimera and DNA. Moreover, nearly all hydrogen bonds between CLFchimera and DNA involved the backbone phosphate groups of DNA, indicating that CLFchimera targets the nucleic acids in a sequence-unspecific manner.

A conformational clustering analysis suggests that CLFchimera mostly performs its activity through the interaction of its two α-helices with DNA. These secondary structure elements, which are stable throughout the simulations, facilitate an optimal presentation of charge density and orientation for the interaction with the phosphate groups. The outcomes of this study provide insight into the structural and dynamic aspects of the interaction between Lactoferrin-derived AMPs and DNA as well as new directions for the design of novel AMPs with intracellular activity.

## Supplementary information

**Additional file 1: Figure S1.** Second replicate: Structural fluctuation analysis. (A) RMSD as function of time; (B) RMSF per residue.

**Additional file 2: Figure S2.** Third replicate: Structural fluctuation analysis. (A) RMSD as function of time; (B) RMSF per residue.

**Additional file 3: Figure S3.** Second replicate: COM distance analysis. (A) COM distances between CLFcin, CLFampin and CLFchimera and DNA along 200 ns. (B) Structures at times t = 0 (cyan) and t = 200 ns (purple): (B1) CLFcin-DNA, (B2) CLampin-DNA, and (B3) CLFchimera-DNA.

**Additional file 4: Figure S4.** Third replicate: COM distance analysis. (A) COM distances between CLFcin, CLFampin and CLFchimera and DNA along 200 ns. (B) Structures at times t = 0 (cyan) and t = 200 ns (purple): (B1) CLFcin-DNA, (B2) CLampin-DNA, and (B3) CLFchimera-DNA.

**Additional file 5: Figure S5.** second replicate: Number of hydrogen bonds with DNA as function of simulation time (200 ns). (A) CLFampin, (B) CLFcin, (C) CLFchimera.

**Additional file 6: Figure S6.** Third replicate: Number of hydrogen bonds with DNA as function of simulation time (200 ns). (A) CLFampin, (B) CLFcin, (C) CLFchimera.

**Additional file 7: Table S1.** The value of hydrogen bond, salt bridge and contacting surface area for three replicates.

**Additional file 8: Figure S7.** Second and Third replicates: Contacting surface area between peptide and DNA along a 200 ns MD simulation.

**Additional file 9: Figure S8**. Second and Third replicates: Estimated binding free energy for the peptide-DNA systems. Calculated with the MM/PBSA method on the 100–200 ns period of one of the simulation replicates.

**Additional file 10: Figure S9**. Second and Third Replicates: Contribution to DNA binding free energies of amino-acid residues in CLFchimera.

**Additional file 11: Figure S10.** Minimum distances during 200 ns simulation.

**Additional file 12: Figure S11.** Second replicate: Number of hydrogen bonds with DNA at different concentrations of CLFchimera, (A) CLFchimera, (B) 2-CLFchimera, (C) 3-CLFchimera, (D) 4-CLFchimera.

**Additional file 13: Figure S12.** Third replicate: Number of hydrogen bonds with DNA at different concentrations of CLFchimera, (A) CLFchimera, (B) 2-CLFchimera, (C) 3-CLFchimera, (D) 4-CLFchimera.

**Additional file 14: Table S2.** Interaction results from MD simulations of CLF-chimera concentration with DNA.

**Additional file 15: Figure S13.** Second and Third replicates: Contacting surface area at different concentrations of CLFchimera.

**Additional file 16: Figure S14.** Second and Third replicates: Contribution of amino-acid residues to DNA-binding energy at different concentrations of CLFchimera.

## Data Availability

Data sharing is not applicable to this article as the datasets that support the findings of this study are included within the article and its Additional files.

## Abbreviations

AMP: Antimicrobial peptides
CLFampin: Camel lactoferrampin
CLFcin: Camel Lactoferricin
COM: Center of mass
CSA: Contacting surface area
DA: Deoxyadenosine
DC: Deoxycytidine
DG: Deoxyguanosine
DNA: Deoxyribonucleic acid
DT: Deoxythymidine
LFampin: Lactoferrampin
LFcin: Lactoferricin
LINCS: Linear constraint solver
MM/PBSA: Poisson-Boltzmann and surface area continuum solvation
PB: Poisson-Boltzmann
PDB: Protein data bank
PME: Particle mesh ewald
RMSD: Root mean square deviation
RMSF: Root-mean-square-fluctuations
SASA: Solvent accessible surface area
SPC: Simple point charge
