# Supplementary material for: Interaction of camel Lactoferrin derived peptides with DNA: a molecular dynamics study
Source: BMC Genomics. 2020 Jan 20;21:60. doi: 10.1186/s12864-020-6458-7 (PMC6971935; doi:10.1186/s12864-020-6458-7)
Supplement: Supplementary file 2 — Additional file 2: Figure S2. Third replicate: Structural fluctuation analysis. (A) RMSD as function of time; (B) RMSF per residue. [file 12864_2020_6458_MOESM2_ESM.pdf]

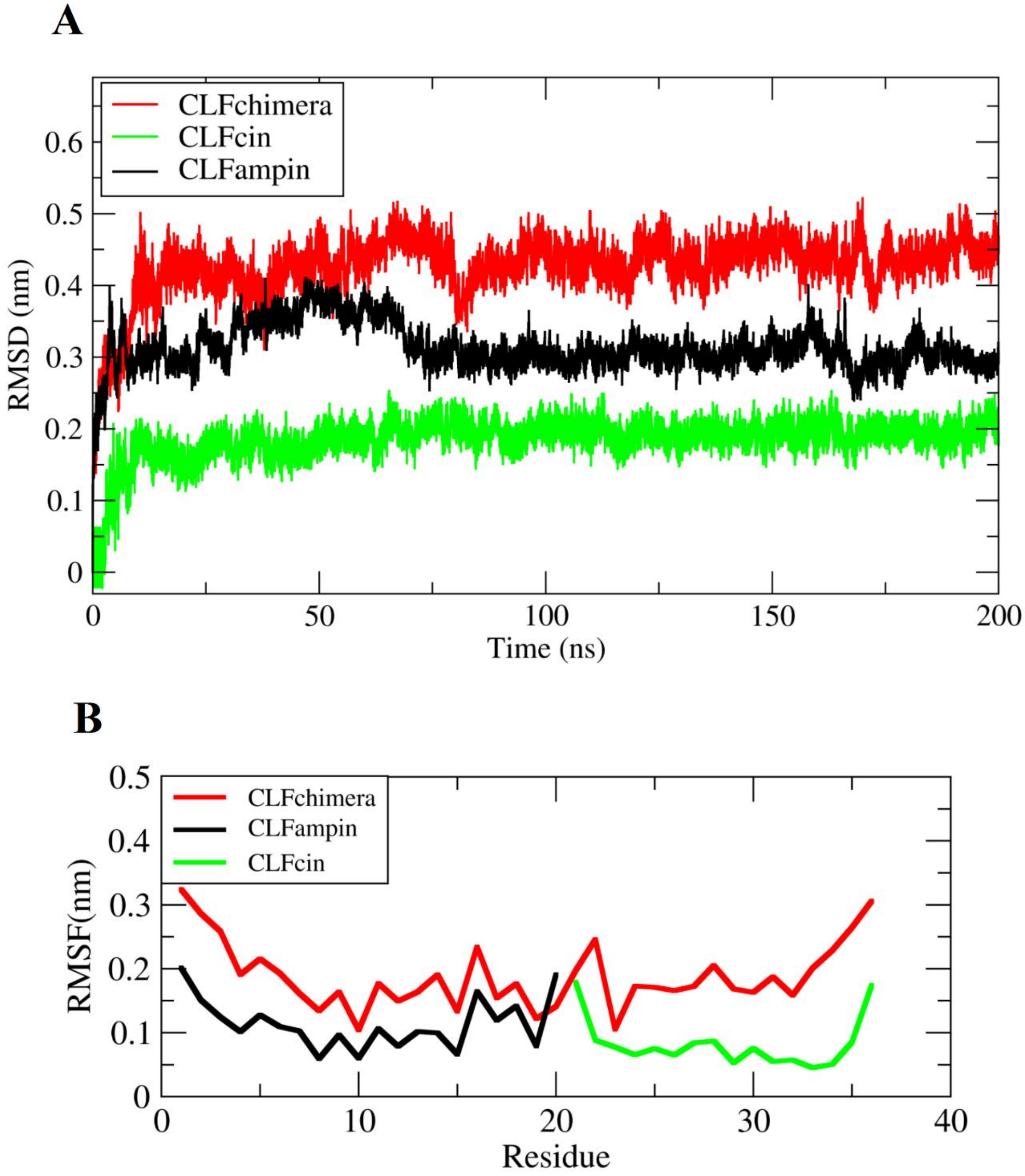

Figure S2. Third replicate: Structural fluctuation analysis. (A) RMSD as function of time; (B) RMSF per residue
