# Supplementary material for: Interaction of camel Lactoferrin derived peptides with DNA: a molecular dynamics study
Source: BMC Genomics. 2020 Jan 20;21:60. doi: 10.1186/s12864-020-6458-7 (PMC6971935; doi:10.1186/s12864-020-6458-7)
Supplement: Supplementary file 4 — Additional file 4: Figure S4. Third replicate: COM distance analysis. (A) COM distances between CLFcin, CLFampin and CLFchimera and DNA along 200 ns. (B) Structures at times t = 0 (cyan) and t = 200 ns (purple): (B1) CLFcin-DNA, (B2) CLampin-DNA, and (B3) CLFchimera-DNA. [file 12864_2020_6458_MOESM4_ESM.pdf]

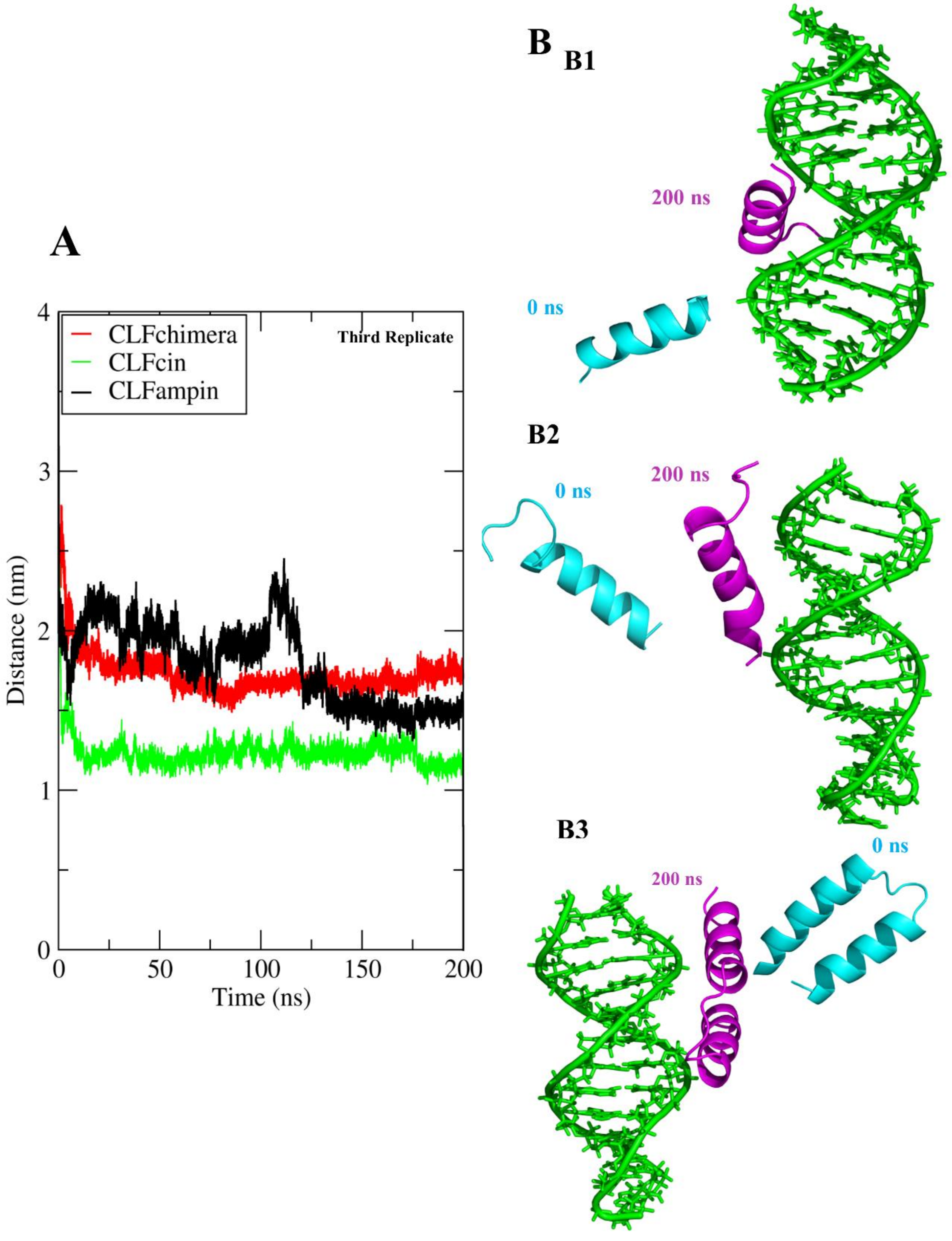

Figure S4. Third replicate: COM distance analysis. (A) COM distances between CLFcin, CLFampin and CLFchimera and DNA along 200 ns. (B) Structures at times t=0 (cyan) and t=200ns (purple): (B1) CLFcin-DNA, (B2) CLampin-DNA, and (B3) CLFchimera-DNA
