# Supplementary material for: Interaction of camel Lactoferrin derived peptides with DNA: a molecular dynamics study
Source: BMC Genomics. 2020 Jan 20;21:60. doi: 10.1186/s12864-020-6458-7 (PMC6971935; doi:10.1186/s12864-020-6458-7)
Supplement: Supplementary file 6 — Additional file 6: Figure S6. Third replicate: Number of hydrogen bonds with DNA as function of simulation time (200 ns). (A) CLFampin, (B) CLFcin, (C) CLFchimera. [file 12864_2020_6458_MOESM6_ESM.pdf]

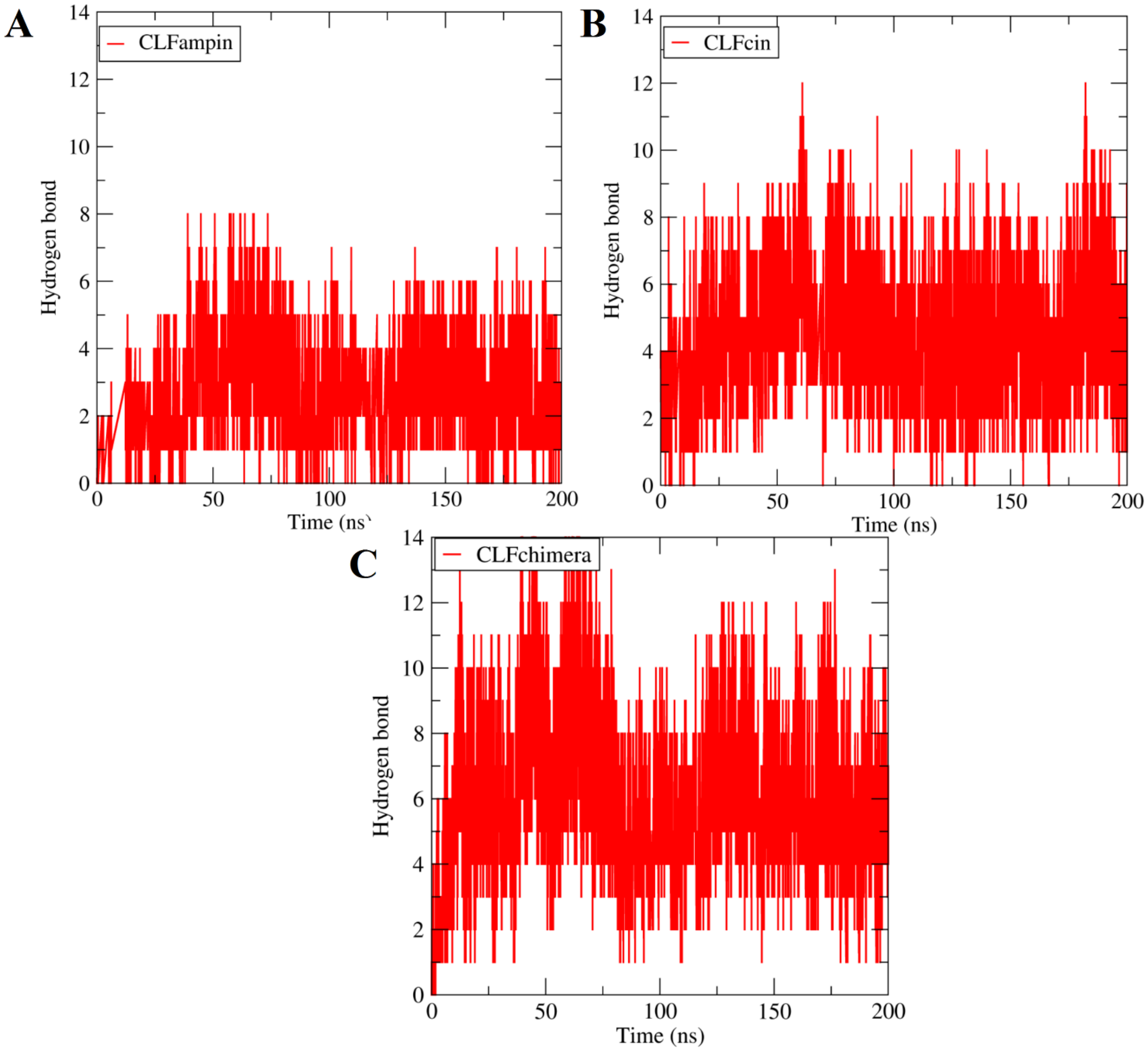

Figure S6. Third replicate: Number of hydrogen bonds with DNA as function of simulation time (200 ns). (A) CLFampin, (B) CLFcin, (C) CLFchimera
