# Supplementary material for: Interaction of camel Lactoferrin derived peptides with DNA: a molecular dynamics study
Source: BMC Genomics. 2020 Jan 20;21:60. doi: 10.1186/s12864-020-6458-7 (PMC6971935; doi:10.1186/s12864-020-6458-7)
Supplement: Supplementary file 7 — Additional file 7: Table S1. The value of hydrogen bond, salt bridge and contacting surface area for three replicates. [file 12864_2020_6458_MOESM7_ESM.pdf]

Table S1. The value of hydrogen bond, salt bridge and contacting surface area for three replicates

		100-200ns		
**Replicates**	**Interactions**	**H-bond**	**Salt bridge**	**CSA**
**RUN1**	CLFcin	5.25	2.91	5.01
	CLFampin	2.88	1.82	4.51
	CLFcimera	5.83	4.15	6.34
	2-CLFchimera	10.12	6.19	11.06
	3-CLFchimera	11.91	6.49	12.91
	4-CLFchimera	12.87	6.98	16.33
**RUN2**	CLFcin	4.20	3.14	4.90
	CLFampin	2.69	1.12	4.60
	CLFcimera	5.77	3.91	5.52
	2-CLFchimera	10.11	5.2	9.13
	3-CLFchimera	11.14	6.21	10.22
	4-CLFchimera	11.95	7.01	12.92
**RUN3**	CLFcin	4.40	3.47	4.81
	CLFampin	2.34	2.1	5.17
	CLFcimera	5.40	4.22	5.92
	2-CLFchimera	8.87	5.98	10.71
	3-CLFchimera	12.22	6.8	12.24
	4-CLFchimera	12.40	7.12	14.41
