# Supplementary material for: Interaction of camel Lactoferrin derived peptides with DNA: a molecular dynamics study
Source: BMC Genomics. 2020 Jan 20;21:60. doi: 10.1186/s12864-020-6458-7 (PMC6971935; doi:10.1186/s12864-020-6458-7)
Supplement: Supplementary file 8 — Additional file 8: Figure S7. Second and Third replicates: Contacting surface area between peptide and DNA along a 200 ns MD simulation. [file 12864_2020_6458_MOESM8_ESM.pdf]

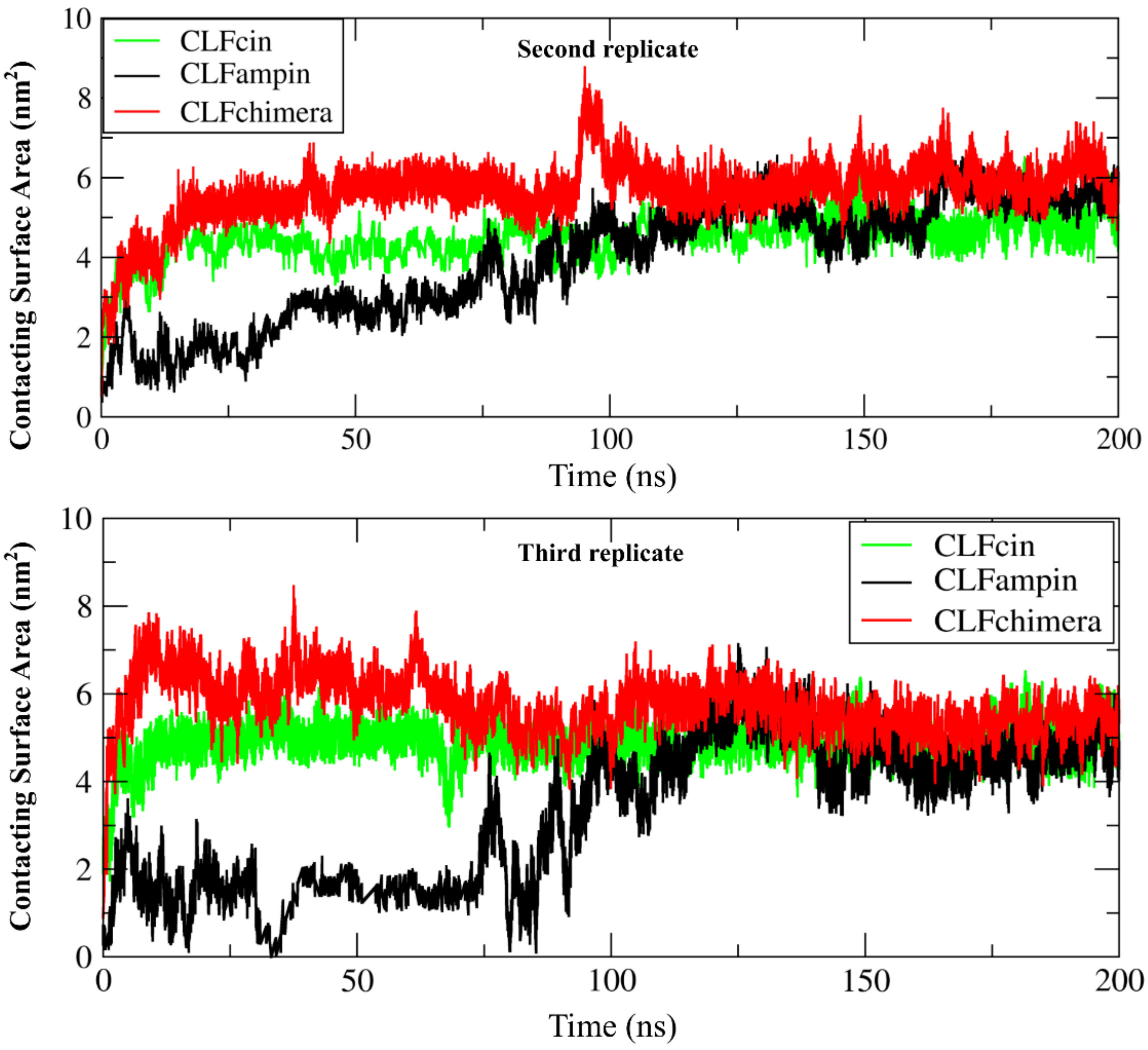

Figure S7. Second and Third replicates: Contacting surface area between peptide and DNA along a 200 ns MD simulation.
