# Supplementary material for: Interaction of camel Lactoferrin derived peptides with DNA: a molecular dynamics study
Source: BMC Genomics. 2020 Jan 20;21:60. doi: 10.1186/s12864-020-6458-7 (PMC6971935; doi:10.1186/s12864-020-6458-7)
Supplement: Supplementary file 9 — Additional file 9: Figure S8. Second and Third replicates: Estimated binding free energy for the peptide-DNA systems. Calculated with the MM/PBSA method on the 100–200 ns period of one of the simulation replicates. [file 12864_2020_6458_MOESM9_ESM.pdf]

Second replicate

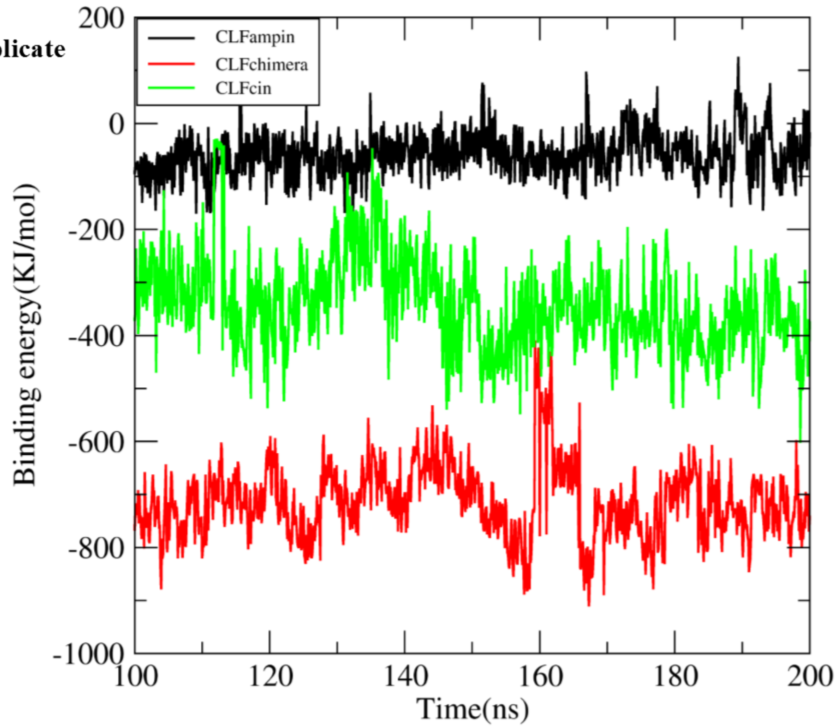

Third replicate

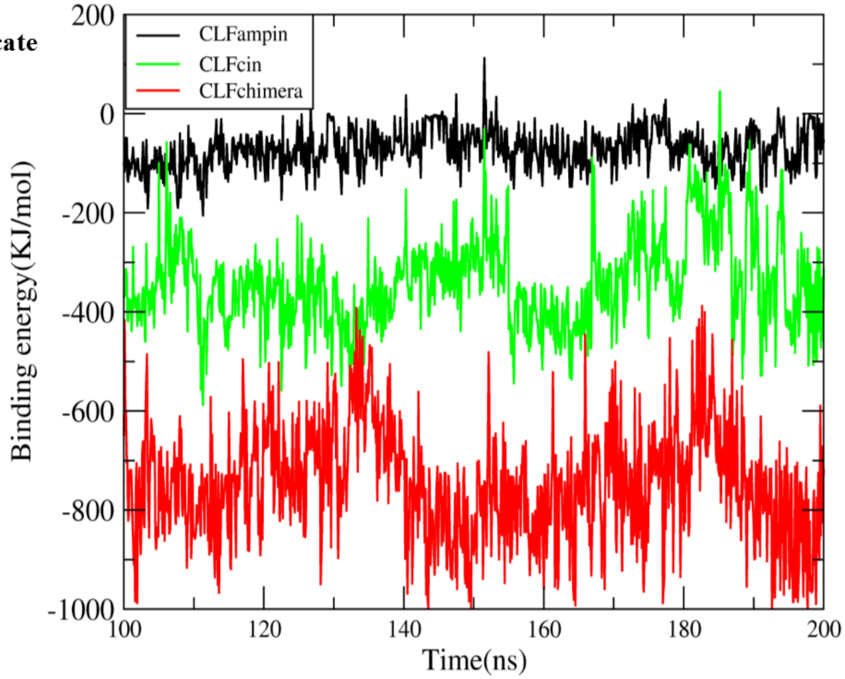

Figure S8. Second and Third replicates: Estimated binding free energy for the peptide-DNA systems. Calculated with the MM/PBSA method on the 100-200 ns period of one of the simulation replicates.
