# Supplementary material for: Interaction of camel Lactoferrin derived peptides with DNA: a molecular dynamics study
Source: BMC Genomics. 2020 Jan 20;21:60. doi: 10.1186/s12864-020-6458-7 (PMC6971935; doi:10.1186/s12864-020-6458-7)
Supplement: Supplementary file 10 — Additional file 10: Figure S9. Second and Third Replicates: Contribution to DNA binding free energies of amino-acid residues in CLFchimera. [file 12864_2020_6458_MOESM10_ESM.pdf]

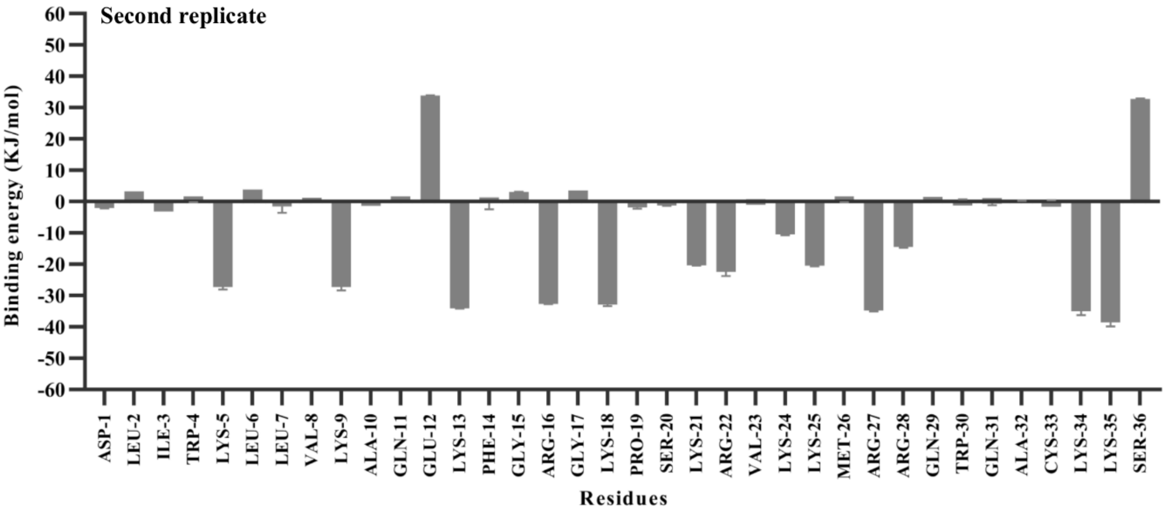

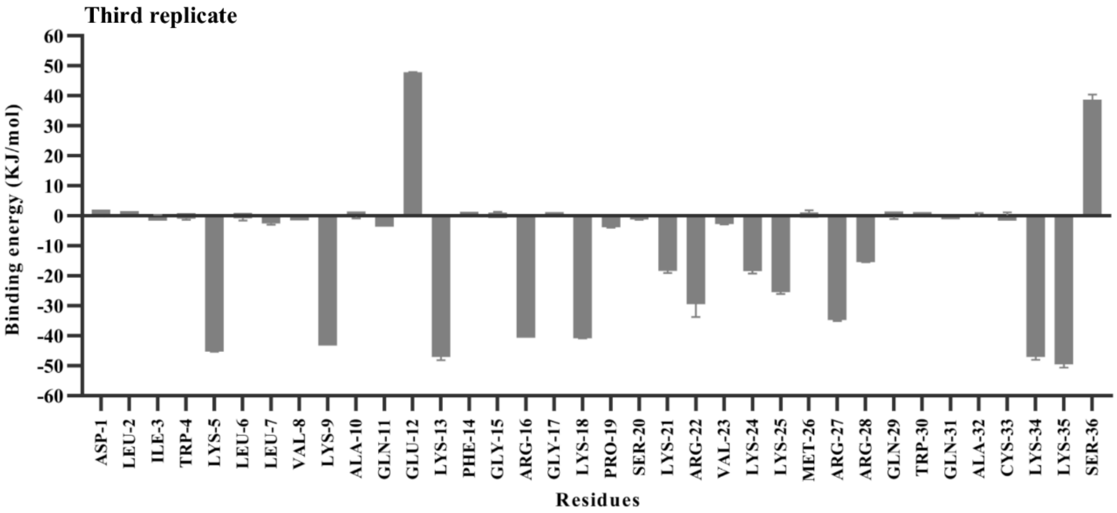

Figure S9. Second and Third Replicates: Contribution to DNA binding free energies of amino-acid residues in CLFchimera
