# Supplementary figures and images for: Interaction of camel Lactoferrin derived peptides with DNA: a molecular dynamics study
Source: BMC Genomics. 2020 Jan 20;21:60. doi: 10.1186/s12864-020-6458-7 (PMC6971935; doi:10.1186/s12864-020-6458-7)

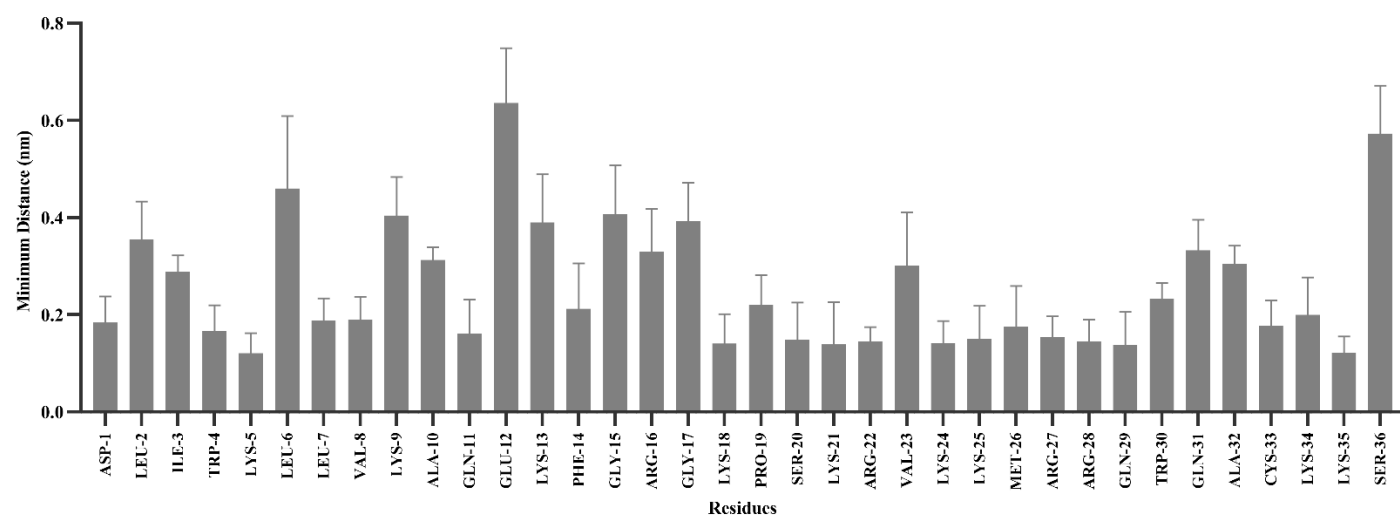

**Figure S10. Minimum distances during 200ns simulation**

Supplement: Supplementary file 11 — Additional file 11: Figure S10. Minimum distances during 200 ns simulation. [file 12864_2020_6458_MOESM11_ESM.pdf]
