# Supplementary material for: Interaction of camel Lactoferrin derived peptides with DNA: a molecular dynamics study
Source: BMC Genomics. 2020 Jan 20;21:60. doi: 10.1186/s12864-020-6458-7 (PMC6971935; doi:10.1186/s12864-020-6458-7)
Supplement: Supplementary file 12 — Additional file 12: Figure S11. Second replicate: Number of hydrogen bonds with DNA at different concentrations of CLFchimera, (A) CLFchimera, (B) 2-CLFchimera, (C) 3-CLFchimera, (D) 4-CLFchimera. [file 12864_2020_6458_MOESM12_ESM.pdf]

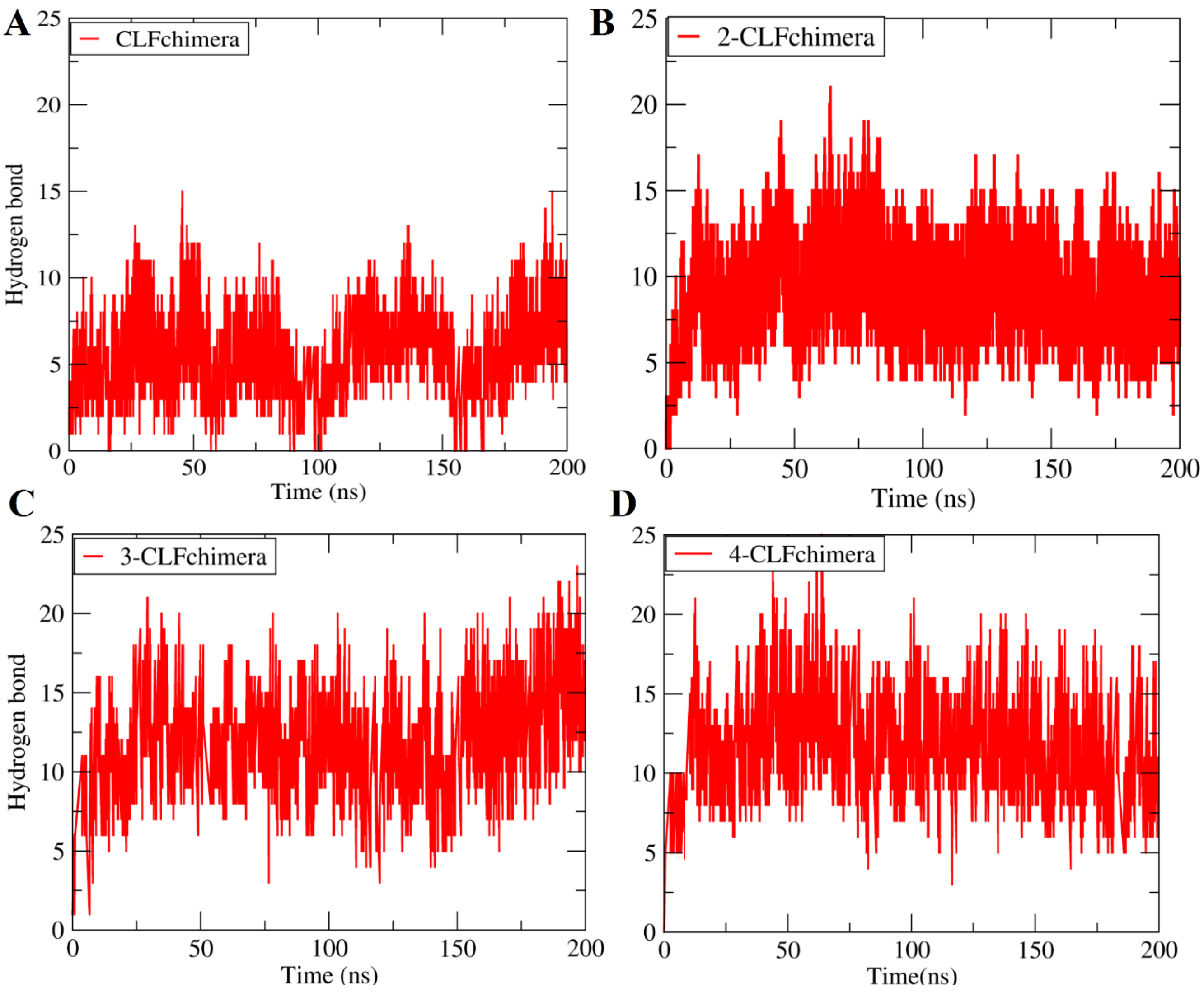

Figure S11, Second replicate: Number of hydrogen bonds with DNA at different concentrations of CLFchimera, (A) CLFchimera, (B) 2-CLFchimera, (C) 3-CLFchimera, (D) 4-CLFchimera.
