# Supplementary material for: Interaction of camel Lactoferrin derived peptides with DNA: a molecular dynamics study
Source: BMC Genomics. 2020 Jan 20;21:60. doi: 10.1186/s12864-020-6458-7 (PMC6971935; doi:10.1186/s12864-020-6458-7)
Supplement: Supplementary file 13 — Additional file 13: Figure S12. Third replicate: Number of hydrogen bonds with DNA at different concentrations of CLFchimera, (A) CLFchimera, (B) 2-CLFchimera, (C) 3-CLFchimera, (D) 4-CLFchimera. [file 12864_2020_6458_MOESM13_ESM.pdf]

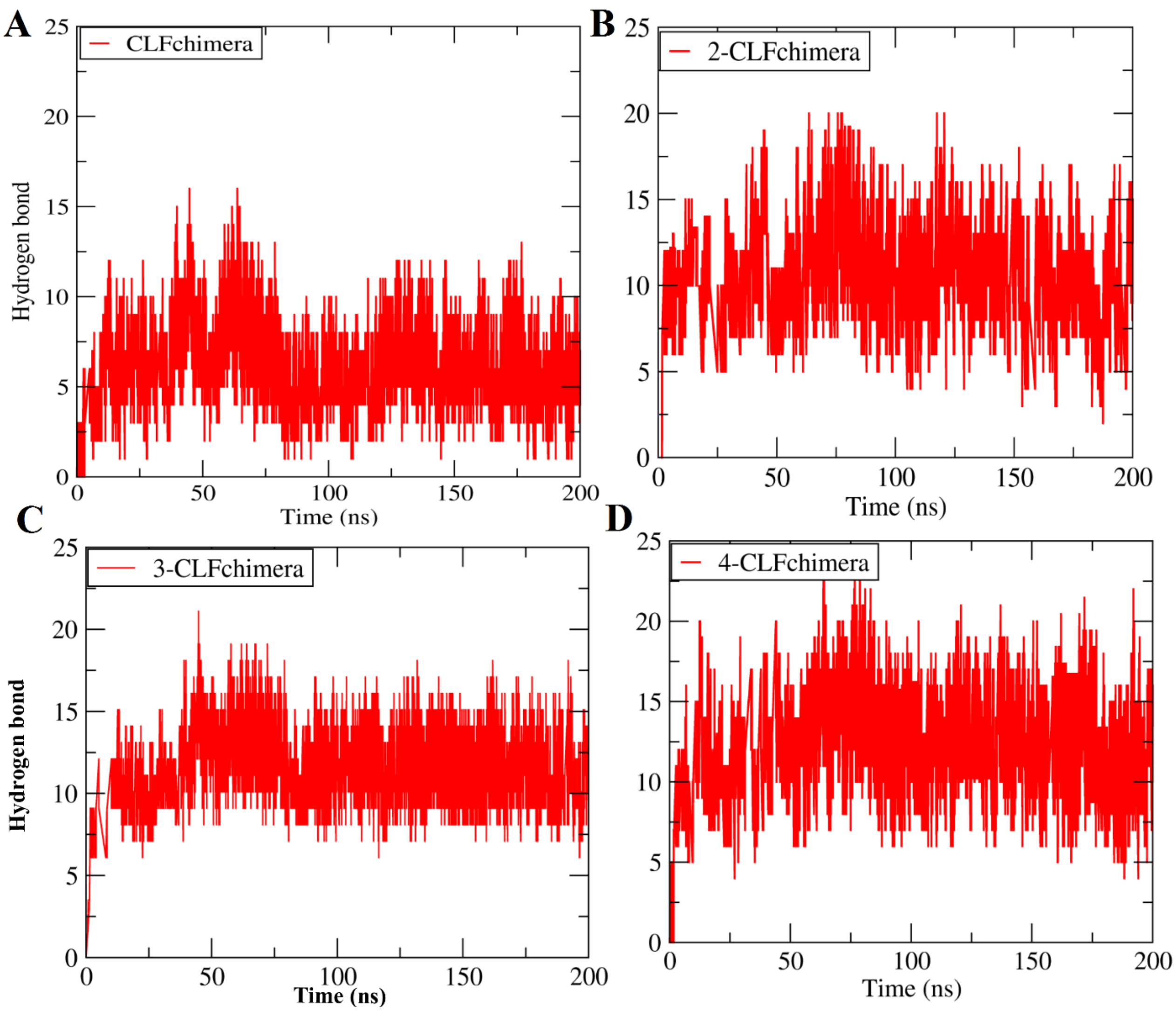


**Figure S12, Third replicate: Number of hydrogen bonds with DNA at different concentrations of CLFchimera,** (A) CLFchimera, (B) 2-CLFchimera, (C) 3-CLFchimera, (D) 4-CLFchimera.
