# Supplementary material for: Interaction of camel Lactoferrin derived peptides with DNA: a molecular dynamics study
Source: BMC Genomics. 2020 Jan 20;21:60. doi: 10.1186/s12864-020-6458-7 (PMC6971935; doi:10.1186/s12864-020-6458-7)
Supplement: Supplementary file 14 — Additional file 14: Table S2. Interaction results from MD simulations of CLF-chimera concentration with DNA. [file 12864_2020_6458_MOESM14_ESM.pdf]

TableS2: Interaction results from MD simulations of CLF-chimera concentration with DNA.

	% Hydrogen bonds to phosphate groups			
simulation	replicates			
	1	2	3	Average
1-CLF-Chimera	100	99.064	98.410	99.158
2-CLF-Chimera	98.011	97.803	99.385	98.39
3-CLF-Chimera	98.041	96.154	97.902	97.36
4-CLF-Chimera	91.042	93.094	98.091	94.35
