# Supplementary material for: Interaction of camel Lactoferrin derived peptides with DNA: a molecular dynamics study
Source: BMC Genomics. 2020 Jan 20;21:60. doi: 10.1186/s12864-020-6458-7 (PMC6971935; doi:10.1186/s12864-020-6458-7)
Supplement: Supplementary file 16 — Additional file 16: Figure S14. Second and Third replicates: Contribution of amino-acid residues to DNA-binding energy at different concentrations of CLFchimera. [file 12864_2020_6458_MOESM16_ESM.pdf]

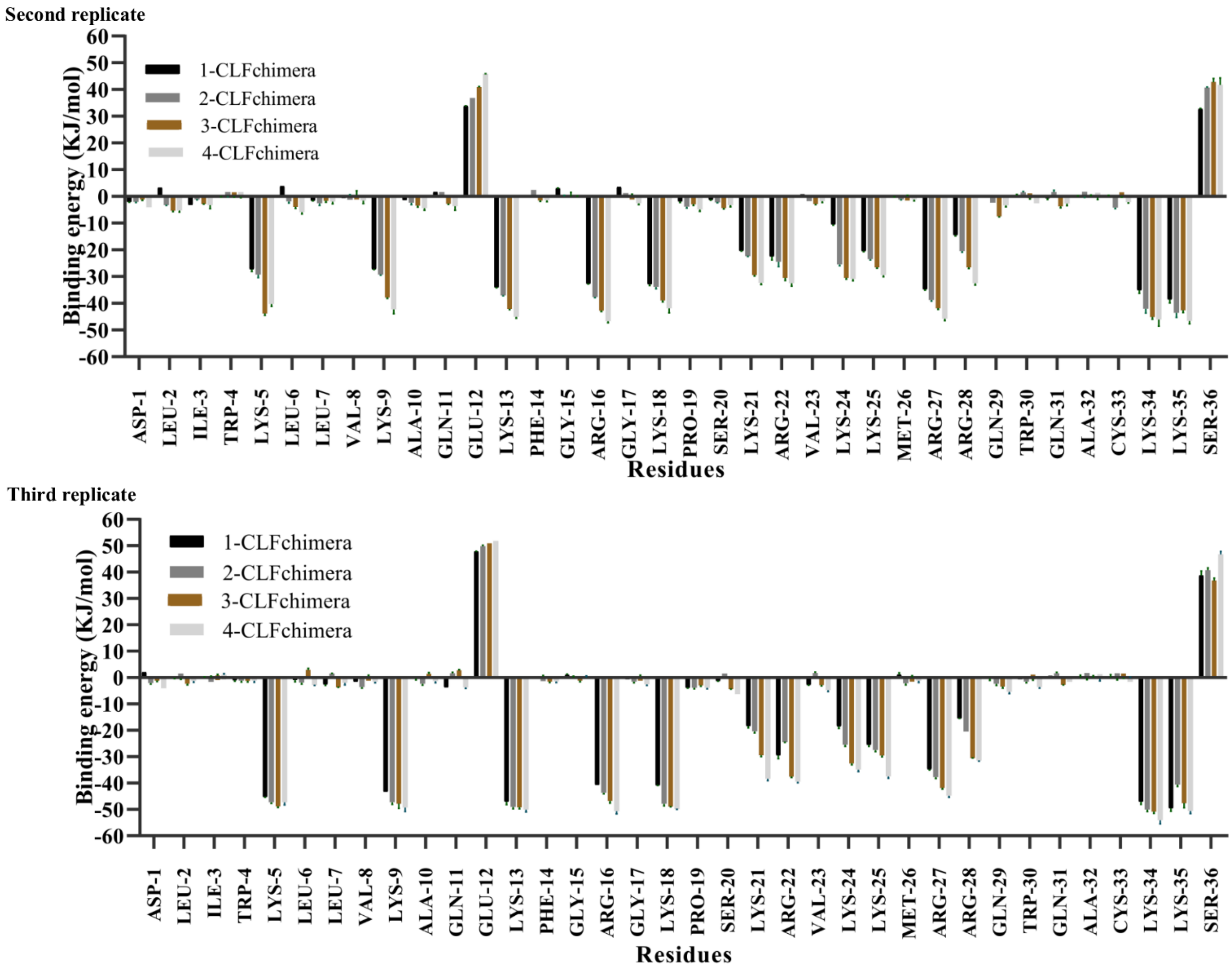

Figure S14.Second and Third replicates: Contribution of amino-acid residues to DNA-binding energy at different concentrations of CLFchimera
